# Socializing One Health: an innovative strategy to investigate social and behavioral risks of emerging viral threats

**DOI:** 10.1186/s42522-021-00036-9

**Published:** 2021-05-14

**Authors:** Karen Saylors, David J. Wolking, Emily Hagan, Stephanie Martinez, Leilani Francisco, Jason Euren, Sarah H. Olson, Maureen Miller, Amanda E. Fine, Nga Nguyen Thi Thanh, Phuc Tran Minh, Jusuf D. Kalengkongan, PREDICT Consortium, Ola Ababneh, Mustafa Ababneh, Jum Rafiah Abd Sukor, Mohd Lutfi Abdullah, Josefina Abedin, Ehab Abu-Basha, Mohamed Ahmed Ali, Junior Beal Akoundze, Joël Akpaki, Sief Addeen Al Hanandeh, Bilal Al Omari, Abdullah Al Shakil, Mohammad Borhan Al-Zghoul, Stephenie Ann Albart, Abdullah Alshammari, Basil H. Amarneh, William Ampofo, Victoria Andrew, Dao Le Anh, Ulaankhuu Ankhanbaatar, Simon Anthony, Ungke Antonjaya, Kidan Araya, Jallah Arku, Norsharina Arshat, Theodore Asigbee, Ohnmar Aung, Joseph Awuni, James Ayukebong, Mohammad Saifullah Mohd Azian, Nor Adilah Aziz, Aminata Ba, Ganzorig Baasan, Ola Bagato, Aboubacar Bamba, Djeneba Bamba, James Bangura, Ariunbaatar Barkhasbaatar, June Barrera, Cale Basaraba, Samuel Bel-Nono, Manjunatha Belaganahalli, Desalegn Belay, Jaber Belkhiria, Ridzki M. F. Binol, Brian Bird, Manisha Bista, Pitu Biswas, Matthew Blake, Linda Boatemaa, Margret Bonason, James Brandful, Joseph Brown, John Brownstein, Alpha Oumar Camara, Mamadi Camara, Salif Camara, Daniel Chai, Debapriyo Chakraborty, Hannah Chale, Ashok Chaudhary, Sokha Chea, Aleksei A. Chmura, Andrew Chow, Carolina Churchill, Abraham Commey, Emmanuel Couacy-Hymann, Julien Kalpy Coulibaly, Michael Cranfield, Wirda Damanik, Batchuluun Damdinjav, Norhidayah Danial, Peter Daszak, Runie David, Patrick Dawson, Ardjouma Dembele, Awa Deme, James Desmond, Aghnianditya Kresno Dewantari, Jasjeet Dhanota, Tapan Dhole, Nguyen Thi Diep, Aristide Dionkounda, Gaye Laye Diop, Kimberly Dodd, Otilia Dogbey, Tumendemberel Dorjnyam, Mireille Dosso, Kalil Doumbouya, Mohamed Idriss Doumbouya, Megan Doyle, Simone Dramou, Tracy Drazenovich, Dang Duc Anh, Bach Duc Luu, Prateep Duengkae, Vu Trong Duoc, Tran Nhu Duong, Veasna Duong, Huda Dursman, Philippe Dussart, Shusmita Dutta, Tan Jun Ee, Abel Ekiri, Amira S. El Rifay, Rabeh El Shesheny, Ahmed N. El Taweel, Zena Babu Emmanuel, Jonathan H. Epstein, Jason Euren, Tierra Smiley Evans, Alaa Fahmawi, Simeon Fahn, Yasha Feferholtz, Jinnat Ferdous, Amanda Fine, Meerjady Sabrina Flora, Leilani Francisco, Lem Fui Fui, Taylor Gabourie, Millawati Gani, Michael Garbo, Nicole Gardner, Aiah Gbakima, Marie Pelagie Atrou Gbamele, Xingyi Ge, Lee Heng Gee, Brooke Genovese, Alexandra Gibson, Kirsten Gilardi, Martin Gilbert, Amethyst Gillis, Andrew Ginsos, Privat Godji Gnabro, Tracey Goldstein, Mokhtar R. Gomaa, Jules Gomis, Kevin Gonzalez, Zoe Grange, Denise Greig, Michael Grodus, Kpon Kakeuma Romeo Gueu, Leticia Gutierrez, Dan Marcelin Haba, Emily Hagan, Abdul Hai, Suraya Hamid, Daniel K. Harris, Abdul Kadir Abu Hashim, Moushumi Hassan, Quaza Nizamuddin Hassan, Qun He, Thiravat Hemachudha, Helena Henry, Ronald Herbert, Zaidoun Hijazeen, Moukala Ndolo Hilarion, Rebecca Hill, Nguyen Thi Hoa, Paul Horwood, Md. Enayet Hossain, Saddam Hossain, Moh Moh Htun, Ben Hu, Tom Hughes, Vibol Hul, Vo Van Hung, Fatima Hussein, Ghislain Dzeret Indolo, Diah Iskandriati, Ariful Islam, Md. Tarikul Islam, Shariful Islam, Mohd Isnaim Ismail, Zuhair Bani Ismail, Jacques Iyanya, Joel Judson Jaimin, Amara Jambai, Jeffrine Rovie Ryan Japning, Alexter Japrin, Frantz Jean Louis, Titus Joe, Christine K. Johnson, Erica Johnson, Damien Joly, Jyotsna Joshi, Jusuf Kalengkongan, Douokoro Kalivogui, Nenneh Kamara-Chieyoe, Joseph Kamau, Eddy Kambale Syaluha, Ahmed Kandeil, Yagouba Kane, William Karesh, Kandeh Kargbo, Dibesh Karmacharya, Novie Kasenda, Ghazi Kayali, Ahmed S. Kayed, Rudovick Kazwala, Changwen Ke, Lucy Keates, Nigatu Kebede, Bouaphanh Khamphaphongphane, Chong Chee Kheong, Christopher Kilonzo, Ma-Sue Koffa, Amos G. Kollie, Marcel Sidiki Kondiano, Michel Koropo, Valere Kouamé Kouakou, Eugene Kouassi Koffi, Mariam Kourouma, Abdoulaye Ousmane Koutate, Citra Livi Kowel, Hermann Assemien Krou, Charles Kumakamba, Tina Kusumaningrum, Omnia Kutkat, François Lamah, Nguyen Thi Lan, Jennifer Lane, Christian Lange, Emmanuel Larmouth, Alice Latinne, Anne Laudisoit, Joseph Diffo Le Doux, Elizabeth Leasure, Katherine Leasure, Mat LeBreton, Jimmy Lee, Helen Lee, Mei Ho Lee, Amara Leno, Hongying Li, Eliza Liang, Neal Liang, Dorothy Lim, W. Ian Lipkin, Jun Liu, Modou Moustafa Lo, Leonoris Lojivis, Nguyen Van Long, Ashley Lucas, Jean Paul Lukusa, Victor Lungay, Shongo Lushima, Julius Lutwama, Wenjun Ma, Catherine Machalaba, Grace Maganga, Walter Simon Magesa, Sara H. Mahmoud, Maria Makuwa, Asha Makweta, Abdullah Al Mamun, Prajwol Manandhar, Patarapol Maneeorn, Harjeet Mann, Pe Bhele Maomy, Victorine Maptue, Stephanie Martinez, Alice Mathew, Yanne Vanessa Mavoungou, Min Thein Maw, Jonna Mazet, Placide Mbala, Emmanuel Mbuba, Eric Mbunwe, David McIver, Emma Mendelsohn, Valchy Bel-Bebi Miegakanda, Maureen Miller, Phan Quang Minh, Happy Mkali, Yassmin Moatasim, Jean Vivien Mombouli, Corina Monagin, Diego Montecino-Latorre, Arsene Mossoun Mossoun, Ahmed Mostafa, Moctar Mouiche, Romain Bagamboula Mpassi, Alphonce Msigwa, Antoine Mudakikwa, Laura Benedict Mugok, Prime Mulembakani, Suzan Murray, Fakhrul Hatta Musa, Pacifique Musabimana, Samson Mutura, Tunu Mwamlima, Mwokozi Mwanzanilla, Tin Tin Myaing, Theingi Win Myat, Aung Myo Chit, Magassouba N’faly, Manzan Jean N’Guettia, Anatole N’télo, Sylivia Nakimera, Vu Sinh Nam, Rajindra Napit, Senthilvel K. S. S. Nathan, Isamara Navarrete-Macias, Kortu M. Ndebe, Amadou Ndiaye, Daouda Ndiaye, Yohannes Negash, Nguyen Thi Thanh Nga, Ipos Ngay, Pham Thi Bich Ngoc, Fabien Niama, Rock Aimé Nina, Schadrack Niyonzima, Felix Nkom, Cynthia Nkoua, Noorliza Noordin, Rachmitasari Noviana, Julius Nwobegahay, Julius Nziza, Daniel O’Rourke, Tammie O’Rourke, Evangeline Obodai, Ricky Okello Okwir, Kevin Olival, Sarah Olson, Onkirotin Dionne Olva, Victoria Ontiveros, Fernandes Opook, Joko Pamungkas, Chandrawathani Panchadcharam, Pranav Pandit, Henri-Joseph Parra, Tran Minh Phuc, Nguyen Thanh Phuong, Jackson Y. Poultolnor, Saman Pradhan, Eunah Cho Preston, Mathieu Pruvot, Dulam Purevtseren, Dhiraj Puri, Le Tin Vinh Quang, Novie Rachmitasari, Kaisar Rahman, Mizanur Rahman, Mohammed Ziaur Rahman, Mustafizur Rahman, Diana Ramirez, Nistara Randhawa, Samita Raut, Joseph Rosario, Albert Ross, Noam Ross, Melinda Rostal, Pamela Roualdes, Eddy Rubin, Aftab Uddin Rumi, Christina Rundi, Melkor Sackie, Dodi Safari, Zikankuba Sajali, Sandra G. Samuels, Mathias Sango, Ammar Rafidah Saptu, Suryo Saputro, Daniel N’guessan Saraka, Alvis A. Sartee, Sia Alida Sayandouno, Karen Saylors, Mame Cheikh Seck, Victoria Sedor, Shahanaj Shano, Ajay Narayan Sharma, Velsri Sharminie, Mahmoud M. Shehata, Gafur Sheikh, Zhengli Shi, Enkhtuvshin Shiilegdamba, Bishwo Shrestha, Rima Shrestha, Mohammed Sidibey, Soubanh Silithammavong, Daniel Simon, Emilly Sion, Symphorosa Sipangkui, Frankie Thomas Sitam, Brett Smith, Bridgette Smith, Woutrina Smith, Batsikhan Sodnom, Benard Ssebide, Maria Suleiman, Ava Sullivan, Nur Amirah Sungif, Richard Suu-Ire, Mouhamed Sy, Jean Michel Takuo, Hani Talafha, Ubald Tamoufe, Emmanuel Tetteh, Aung Than Toe, Lanash Thanda, Ngo Thanh Long, Wai Zin Thein, Watthana Theppangna, Nguyen Duc Thinh, Hoang Bich Thuy, Nguyen Thu Thuy, Eri Togami, Moise Bendoua Tolno, Kevin Tolovou, Rahmat Topani, Alexandre Tremeau-Bravard, Ian Trupin, Jean Claude Tumushime, Kyaw Yan Naing Tun, Joseph Turay, Helal Uddin, Marcela Uhart, Nicole Ureda, Marc Valitutto, Khebir Verasahib, Megan Vodzak, Supaporn Wacharapluesadee, Mohammad Yuery Wazlan Abdul Wahad, Brooke Watson, Heather Wells, Allison White, Anna Willoughby, Ageng Wiyatno, David Wolking, Xinglou Yang, Lim Ming Yao, Sayon Yombouno, Cristin Young, Carlos Zambrana-Torrelio, Zahidah Izzati Zeid, Ghadeer Zghoul, Libiao Zhang, Yunzhi Zhang, Guangjian Zhu, Dawn Zimmerman, Daba Zoumarou, Alonso Aguirre, Luis Aguirre, Mark-Joel Akongo, Erika Alandia Robles, Laurentius Ambu, Simon Anthony, Glenda Ayala Aguilar, Luis Barcena, Rosario Barradas, Misliah Mohamad Basir, Tiffany Bogich, Gerard Bounga, Philippe Buchy, David Bunn, Denis Byaruba, Ken Cameron, Dennis Carroll, Nancy Cavero, Manuel Cespedes, Xiaoyu Che, Charles Chiu, Aleksei Chmura, Kimashalen Chor, Andrew Clements, Luz Dary Acevedo, Angelica de Almeida Campos, Micaela De La Puente, Xavier de Lamballerie, Catia de Paula, Eric Delwart, Joseph Diffo Le Doux, Catherine Doyle-Capitman, Edison Durigon, Joseph Fair, José R. Ferrer-Paris, Pierre Formenty, Isabel Galarza, Joel Garcia, Benoit Goossens, Gilda Grard, Zoe Greatorex, Laurie Harris, Peta Hitchens, Mei Ho, Parviez Hosseini, Tom Hughes, Samath In, Volga Iñíguez, Diah Iskandriati, Ariful Islam, Jacques Iyanya, Komal Jain, Abd. Aziz Jamaluddin, Jeffrine Rovie Ryan Japning, Christine Johnson, Damien Joly, Kate Jones, Priscilla Joyner, Serge Kaba, Eddy Kambale, William Karesh, Dibesh Karmacharya, Abdulhameed Kataregga, Rudovick Kazwala, Changwen Ke, Terra Kelly, Chong Chee Kheong, Kongsy Khammavong, A. Marm Kilpatrick, Samsir Laimun, Mat LeBreton, Helen Lee, Mei-Ho Lee, Jimmy Lee, Eric LeRoy, Jordan Levinson, Marc Levy, Eliza Liang, Neal Liang, Rolando Limachi, W. Ian Lipkin, Elizabeth Loh, Leonoris Lojivis, Linda J. Lowenstine, José Luis Mollericona, Shongo Lushima, Wenjun Ma, Catherine Machalaba, Ruth Maganga, Maria Makuwa, Joseph Malakalinga, Patarapol Maneeorn, Melissa Manhas, Pete Marra, Alice Mathew, Jonna Mazet, Rachael Mbabazi, Placide Mbala, Rodrigo Medellín, Patricia Mendoza, Sireeda Miller, Flavia Miranda, Megan Mitchell, Ramlan Mohamed, Debbie Mollard, Corina Monagin, Stephen Morse, Wivine Mouellet, Isabel Moya, Antoine Mudakikwa, Laura Benedict Mugok, Prime Mulembakani, Yovanna Murillo, Kris Murray, Suzan Murray, Jean-Jacques Muyembe Tamfum, Sylivia Nakimera, Fernando Nassar, Sen Nathan, Isamara Navarrete-Macias, Ipos Ngay, Schadrack Niyonzima, Felix Nkom, Noorliza Noordin, Rachmitasari Noviana, Olivier Nsengimana, Julius Nziza, Lucy Ogg Keatts, Rafael Ojeda-Flores, Ricky Okwir Okello, Kevin Olival, Sarah Olson, Alain Ondzie, Daniel O’Rourke, Tammie O’Rourke, Joko Pamungkas, Janusz Paweska, Alisa Pereira, Victoria Pereira, Alberto Perez, Jocelyn Perez, Simorn Phon, Diana Ramirez, Patricia Reed, Dan Rejmanek, Oscar Rico, Rosario Rivera, Monica Romero, Joseph Rosario, Melinda Rostal, Celina Roy, Christina Rundi, Uus Saepuloh, Dodi Safari, Zikankuba Sajali, Karen Saylors, Brad Schneider, Jessica Schwind, Zhengli Shi, Sinpakhome Singhalath, Symphorosa Sipangkui, Frankie Thomas Sitam, Brett Smith, Kristine Smith, Woutrina Smith, Benard Ssebide, Fabiola Suárez, Maria Suleiman, Gerardo Suzan, Jean Michel Takuo, Ubald Tamoufe, Lanash Thanda, Nguyen Thi Thanh Nga, Kate Thomas, Herminio Ticona, Alexandre Tremeau-Bravard, Marcela Uhart, Elizabeth VanWormer, Sandra Villar, Wendy Weisman, Michael Westfall, Chris Whittier, Leanne Wicker, Nathan Wolfe, Angela Yang, Carlos Zariquiey, Shu-Yi Zhang, Baudelaire Zorine Nkouantsi, Zainal Zainuddin, Cara Chrisman, August Pabst, Amalhin Shek, Murray Trostle, Tina Kusumaningrum, Alice Latinne, Joko Pamungkas, Dodi Safari, Suryo Saputro, Djeneba Bamba, Kalpy Julien Coulibaly, Mireille Dosso, Anne Laudisoit, Kouassi Manzan N’guettia Jean, Shusmita Dutta, Ariful Islam, Shahanaj Shano, Mwokozi I. Mwanzalila, Ian P. Trupin, Aiah Gbakima, James Bangura, Sylvester T. Yondah, Dibesh Karmacharya, Rima D. Shrestha, Marcelle Annie Matsida Kamta, Mohamed Moctar Mouliom Mouiche, Hilarion Moukala Ndolo, Fabien Roch Niama, Dionne Onikrotin, Peter Daszak, Christine K. Johnson, Jonna A. K. Mazet

**Affiliations:** 1 Labyrinth Global Health, St. Petersburg, FL USA; 2 One Health Institute, University of California, Davis, One Shields Ave, Davis, CA 95616 USA; 3 EcoHealth Alliance, New York, NY 10001 USA; 4 Henry Jackson Foundation, Bethesda, MD 20817 USA; 5 Metabiota, Inc, San Francisco, CA 94104 USA; 6 Wildlife Conservation Society, Health Program, 2300 Southern Blvd, Bronx, NY 10460-1099 USA; 7 Mailman School of Public Health, Columbia University, New York, NY 10032 USA; 8 The Wildlife Conservation Society, Viet Nam Country Program, Hanoi, Vietnam; 9 Eijkman Institute for Molecular Biology, Jakarta, Indonesia; 10 Primate Research Center and Faculty of Veterinary Medicine, IPB University, Bogor, Indonesia; 11 Institut Pasteur of Côte d’Ivoire, Abidjan, Côte d’Ivoire; 12 Institute of Epidemiology, Disease Control and Research, Dhaka, Bangladesh; 13 Sokoine University of Agriculture, Morogoro, Tanzania; 14 University of Makeni, Makeni, Sierra Leone; 15 Center for Molecular Dynamics Nepal, Kathmandu, Nepal; 16 Ministry of Scientific Research and Innovation, Yaounde, Cameroon; 17 Mosaic, Yaounde, Cameroon; 18 Independent researcher, Brazzaville, Republic of Congo; 19 Laboratoire National de Sante Publique, Brazzaville, Republic of Congo

**Keywords:** Social science research, Behavioral risk, One health, Multi-disciplinary surveillance

## Abstract

In an effort to strengthen global capacity to prevent, detect, and control infectious diseases in animals and people, the United States Agency for International Development’s (USAID) Emerging Pandemic Threats (EPT) PREDICT project funded development of regional, national, and local One Health capacities for early disease detection, rapid response, disease control, and risk reduction. From the outset, the EPT approach was inclusive of social science research methods designed to understand the contexts and behaviors of communities living and working at human-animal-environment interfaces considered high-risk for virus emergence. Using qualitative and quantitative approaches, PREDICT behavioral research aimed to identify and assess a range of socio-cultural behaviors that could be influential in zoonotic disease emergence, amplification, and transmission. This broad approach to behavioral risk characterization enabled us to identify and characterize human activities that could be linked to the transmission dynamics of new and emerging viruses. This paper provides a discussion of implementation of a social science approach within a zoonotic surveillance framework. We conducted in-depth ethnographic interviews and focus groups to better understand the individual- and community-level knowledge, attitudes, and practices that potentially put participants at risk for zoonotic disease transmission from the animals they live and work with, across 6 interface domains. When we asked highly-exposed individuals (ie. bushmeat hunters, wildlife or guano farmers) about the risk they perceived in their occupational activities, most did not perceive it to be risky, whether because it was normalized by years (or generations) of doing such an activity, or due to lack of information about potential risks. Integrating the social sciences allows investigations of the specific human activities that are hypothesized to drive disease emergence, amplification, and transmission, in order to better substantiate behavioral disease drivers, along with the social dimensions of infection and transmission dynamics. Understanding these dynamics is critical to achieving health security--the protection from threats to health-- which requires investments in both collective and individual health security. Involving behavioral sciences into zoonotic disease surveillance allowed us to push toward fuller community integration and engagement and toward dialogue and implementation of recommendations for disease prevention and improved health security.

**Supplementary Information:**

The online version contains supplementary material available at 10.1186/s42522-021-00036-9.

## Background

Globalization has radically catalyzed the everyday movements of people, animals, technology, goods, capital, and services worldwide. While this transformation has been broadly regarded as an economic boon, it has also increased the opportunities for diseases to spread geographically and potentially between species [24, 29]. Land use change, such as the building of roads or cities where once there were forests, creates a chain reaction of ecological, socio-economic, human behavioral, and regional fauna impacts that are believed to be linked to how infectious diseases emerge. Globally, urbanization has led to drastic growth in the density of human populations living in cities, increasing the potential for large infectious disease outbreaks [8, 16]. Per capita meat consumption has rapidly expanded over the last half century, driving the development of high-density livestock operations that provide opportunities for large-scale animal disease outbreaks [21]. Constant demand for cropland and grazing land, as well as aggressive resource extraction, has resulted in drastic environmental transformations, including habitat destruction, forest encroachment, and interspecies mixing [2]. Zoonotic diseases – those with an animal host or reservoir – are responsible for some of the most impactful and devastating outbreaks in recent years. Seventy-five percent of emerging infectious diseases (EIDs) are zoonotic in origin, including Ebola, Influenza A strains H5N1 and H9N2, Hantaviruses, and human sleeping sickness [15, 25]. One Health, an approach that recognizes human, animal, and environmental health as linked, has proven valuable in recent emerging infectious disease research and surveillance efforts and is representative of trends in the transmission of pathogens across species [4].

### One Health Theory

The concept of One Health is a revitalization and expansion of the concept of One Medicine, developed in the 1970s by Calvin Schwabe to recognize the inextricable interconnection of humans and animals in the domains of nutrition, livelihood, and health. In the 2000s, the concept of One Health was adopted to further broaden the concept to include ecosystem health – including the influence of climate, plants, and wildlife on global health [30]. International organizations, including FAO, OIE, WHO, and The World Bank, soon codified a One Health strategy to guide research and capacity development efforts towards the prevention, detection, and response of infectious diseases [6]. One Health is defined as “a collaborative, multisectoral, and transdisciplinary approach... with the goal of achieving optimal health outcomes recognizing the interconnection between people, animals, plants, and their shared environment [5]".

While the inclusion of the environment in One Health thinking has greatly increased our ability to tackle today’s complex health problems (driven by climate, mobility, and land-use changes), a growing number of scholars argue that resulting One Health work has prioritized the study of “natural” ecological systems over “social” systems [22, 28, 30], as domesticated animals, plants, and wildlife are as much a “part of the environment of humans” as they are a “part of the social systems of humans” [30]. This dynamic, interdependent vision is similar to the biocultural conservation principle that suggests that interventions must be tailored to the social-ecological context and that different worldviews and knowledge systems must be incorporated into conservation planning [11]. Without a nuanced understanding of specific human activities, “how, where and when people interact with animals,” it is impossible to understand the actual risk for zoonotic spillover events [28]. To adequately understand human activity as an integrated part of the environment, One Health teams should strive to include professionals from disciplines like anthropology, economics, political science, psychology, and sociology [22].

### Understanding human behavior through the Ebola lens

The imperative to understand human activity in the context of zoonotic disease outbreaks was perhaps best exemplified during the 2014 West Africa Ebola epidemic, where dysfunctional health systems, denial of the existence of Ebola, and burial practices involving contact with the deceased exacerbated containment of the outbreak [14]. Following recent EVD outbreaks in DRC and Uganda, the behavioral sciences – medical anthropology in particular – have made a critical contribution to understanding the social dynamics of zoonotic disease emergence and spread, as well as developing effective response interventions for disease control [9, 26]. In particular, the WHO’s Ebola Strategy guidelines clearly articulate several critical contributions medical anthropology can have towards outbreak management [27]. First, such research contributes towards “better knowledge of disease transmission chains,” identifying behavioral mechanisms that may be perpetuating spread, such as forms of wildlife contact, exposure to infected medical items, or burial practices. Second, the behavioral sciences can identify “psychologically, socially, and culturally diverse behaviors of local populations” and propose appropriate interventions. By understanding the culturally-specific context and meaning of behaviors driving disease transmission, response efforts can react faster and design more culturally appropriate interventions that are acceptable to the populations being affected. In addition, contributions from social scientists can help to identify rumors, fears, and misinformation that may be amplifying risks for transmission. These contributions can help guide the development of “empathetic approaches” to outbreak response and disease control, striving to engage the participation of affected communities to develop sustainable interventions, as opposed to “coercive approaches” that are largely indifferent to the needs and opinions of communities. A major global health security lesson learned from the West Africa Ebola epidemic was that to strengthen a population’s ability to protect themselves we must better understand how certain behaviors put people at risk, and what changes we can make to mitigate that risk. Being able to communicate how human/animal interactions facilitate the emergence of wildlife pathogens in human populations to public health decision-makers and advocating for behavioral change communication, education, and prevention efforts can improve compliance with and the effectiveness of medical interventions and public health efforts.

## Approach

### Using social sciences to understand spillover risk before emergence

While especially critical in outbreak scenarios, the contributions of the behavioral sciences are equally important prior to disease emergence, as they can improve our understanding of the risks associated with pathogen spillover and spread, and can inform strategies and interventions for risk reduction and mitigation. Quantitative modeling approaches have been used to extrapolate data to help understand pathogen-host dynamics and estimate outbreak frequency and severity, as seen in recent disease hotspot mapping [1] and current research exploring high-risk human-animal interfaces [12]. Human behaviors are complex, dynamic, and highly contextual and are influenced by a myriad of socio-cultural factors that elude traditional disease modeling methods [3, 13]. A multidisciplinary approach to exploring the social dimensions and human behaviors associated with disease transmission is fundamental to more holistically understanding the conditions and circumstances in which zoonotic diseases emerge and spread.

#### The Emerging Pandemic Threats program

In an effort to strengthen global capacity to prevent, detect, and control infectious diseases in animals and people, the United States Agency for International Development’s (USAID) Emerging Pandemic Threats (EPT) program funded several projects to develop regional, national, and local One Health capacities for early disease detection, rapid response, disease control, and risk reduction [15]. From the outset, the EPT approach was inclusive of social science research methods designed to understand the contexts and behaviors of communities living and working at human-animal-environment interfaces considered high-risk for virus emergence. The purpose was to shed light on the social dimensions of zoonotic disease transmission and identify potential intervention strategies for prevention and risk reduction. From 2009 to 2014, EPT’s PREVENT project focused on formative research intended to identify risky behaviors, attitudes, and practices in the Congo Basin and Southeast Asia and worked to identify and characterize vulnerable populations, and the high-risk behaviors and practices for disease transmission from animals to humans. At the same time, the USAID EPT PREDICT project developed a global consortium to strengthen capacity for surveillance and early detection of virus threats from wildlife and to identify high-risk areas and human-animal interfaces for virus spillover, amplification, and spread for targeted surveillance, monitoring, prevention, and control efforts. Working with partners in over 20 countries, PREDICT teams collected samples for virus testing from more than 56,000 animals and detected thousands of unique viruses in what is considered the largest virus detection and discovery effort to date [17].

#### PREDICT 2

**Fig. 1:**
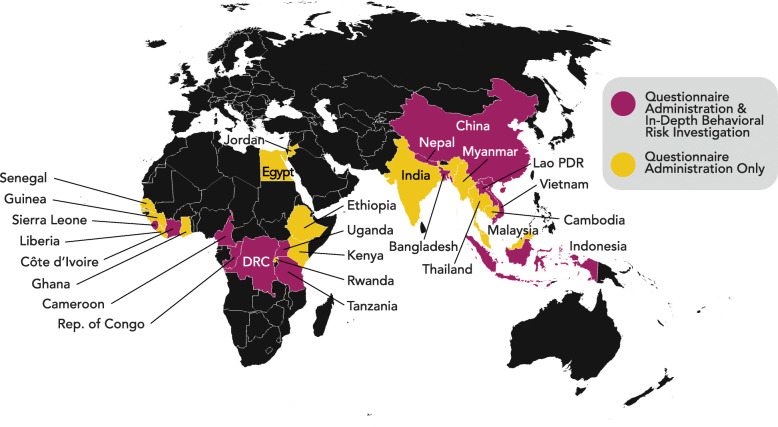
PREDICT behavioral risk investigations. Twenty-seven out of PREDICT’s 28 participating countries implemented questionnaires for quantitative analysis; the exception was Mongolia, which focused exclusively on Influenza A surveillance in wild birds. 13 countries conducted qualitative behavioral risk investigations

Building on this foundation, USAID’s EPT program funded another 5-year investment to strengthen health system capabilities for improved zoonotic disease prevention, detection, and response. In 2014, this second phase of the PREDICT project was launched in Africa, South and Southeast Asia, and East Asia with a revised One Health surveillance strategy reliant on the concurrent sampling of animals and people in identified at-risk interfaces for virus emergence. This new scope included an expanded emphasis on understanding behavioral risks along with data collection, synthesis, and aggregation on biological and ecological risks at these interfaces. PREDICT’s behavioral risk strategy was implemented in 27 countries from 2014 to 2019 (Fig. 1), and was adapted to host country contexts and specific human-animal-environment interfaces, yet data collection was standardized globally to enable cross-country, regional, and ultimately global comparisons [20].

**Fig. 2:**
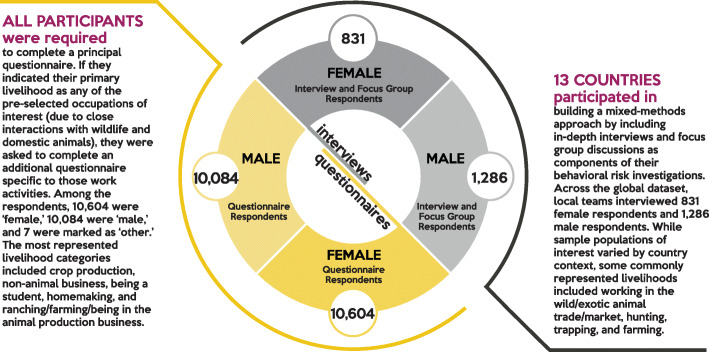
Implementation of questionnaires, ethnographic interviews, and focus group discussions (FGDs)

In each country, a structured quantitative questionnaire was administered whenever a human sample was collected. This 57-item questionnaire covered demographics, travel, hygiene, self-reported illness history, indirect and direct contact with domestic and wild animals, and knowledge, attitudes, and behaviors related to animals and animal meats and byproducts. In addition to the core questionnaire, 10 focused occupational modules were administered based on a participant’s reported occupation or primary livelihood in the past year. A separate questionnaire, developed to address the unique context of the countries affected by the 2014 West Africa Ebola epidemic under PREDICT’s Ebola Host Project (a targeted effort to identify host species for ebolaviruses) was administered in Guinea, Liberia, and Sierra Leone. Over the course of the project, over 20,000 individuals were enrolled and completed questionnaires in these 27 countries (Table 1 and Fig. 2).

**Table 1:** Global summary of behavioral data collection

Country	# Surveys administered^a^	# Interviews conducted
Bangladesh	1,106	102
Cambodia	1,803	---
Cameroon	651	292
China	718	172
Côte d'Ivoire	434	199
Dem. Rep. of the Congo	906	264
Egypt	1,097	---
Ethiopia	313	---
Ghana	641	---
Guinea	335^b^	---
India	65	---
Indonesia	896	125
Jordan	1,085	---
Kenya	327	---
Lao PDR	234	22
Liberia	585^b^	---
Malaysia	1,400	---
Myanmar	708	---
Nepal	2,048	109
Rep. of Congo	23	108
Rwanda	400	---
Senegal	824	---
Sierra Leone	588^b^	179
Tanzania	1,172	402
Thailand	678	---
Uganda	428	66
Vietnam	1,230	77
Total # of individuals enrolled	20,695	2,117

^a^Surveys administered using PREDICT’s standard questionnaire as part of the project’s human surveillance and sampling scope

^b^Surveys administered using a separate targeted questionnaire designed for countries affected by the 2014 West Africa Ebola epidemic

### In-depth behavioral risk investigations

As capacity, time, and funding allowed, each country team could elect to incorporate PREDICT’s qualitative research strategy into behavioral risk investigations. Qualitative tools were designed to complement the standardized questionnaire, and 13 countries worked collaboratively to implement this mixed methods approach. These methods were first used to collect formative baseline data intended to inform the development and rollout of the standardized questionnaire; later they were employed to either continue exploratory work or follow up on preliminary baseline findings and finally, after evaluating preliminary data and insights, to refocus on the identification of intervention and risk reduction strategies. The countries that opted to include qualitative research in their behavioral risk investigations did so due to locally relevant high-risk interfaces that warranted deeper investigation into their contexts and socio-cultural dynamics. Country teams decided on where to target qualitative efforts and how to engage community gatekeepers and key informants who could facilitate access. In DR Congo and Cameroon, for example, investigations focused on wild animal ‘bushmeat’ markets, and teams conducted ethnographic interviews to better understand the market dynamics and behavioral and exposure risks related to key taxa in the bushmeat value chain. In Vietnam, the focus was on wildlife farming practices and biosecurity measures. In the 13 countries implementing this qualitative behavioral risk scope, more than 2,000 individuals were enrolled in ethnographic interviews and focus group discussions, and interviews were transcribed and translated (as necessary) for coding and analysis (Table 1).

### Building behavioral sciences into one health surveillance

By design, the PREDICT Consortium integrated global expertise from the conservation, veterinary medicine, public health, and social science communities to develop collaborative and multidisciplinary approaches for the early detection of virus threats and the development of disease control and prevention recommendations. Data collection tools were collaboratively designed to address ecological risks for emergence (using a standardized observational tool) and socio-behavioral risks (using a standardized questionnaire with the option of conducting additional in-depth behavioral risk investigations through ethnographic interviews and focus group discussions). Standard operating procedures and training materials were developed to assure standardization of the strategy through the life of the project [19]. Once approved by US and host country Institutional Review Boards and ethics committees, the strategy and tools were put into action with partners across all project countries.

At the country level, personnel were identified by local partners to lead the behavioral risk scope and teamed up with Consortium partners for training and mentorship. Because of differences in personnel background, the training and mentorship plan was structured to introduce the basics of social science methodology for rapid onboarding while also diving deep into the PREDICT strategy and behavioral risk tool kit using a combination of lecture, discussion, and hands-on experiential learning. Training covered techniques for successful community engagement and outreach; how to conduct interviews using the questionnaire; ethnographic methods and techniques for leading effective qualitative interviews and focus group discussions; data management, coding, and analysis; and strategies for sharing project findings and communicating risk reduction strategies.

**Fig. 3:**
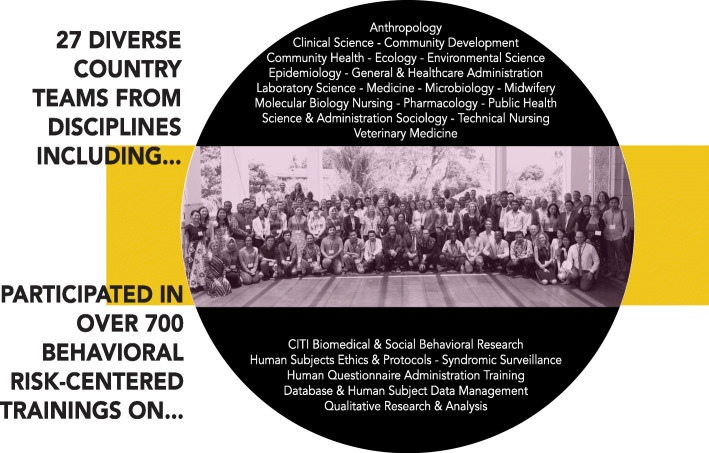
Supporting the Creation of a Global Behavioral Risk Cadre. Bringing together a transdisciplinary team of scientists and practitioners was central to the human behavioral risk surveillance arm of PREDICT. Representing myriad disciplines, local behavioral risk teams were provided with trainings specifically centered on the foundations needed to successfully conduct behavioral risk investigations

During implementation, trained behavioral risk personnel joined teams comprised of local professionals from diverse disciplines (Fig. 3). Teams worked together to engage at-risk communities and conduct behavioral investigations, while animal and public health professionals led One Health surveillance and sampling efforts. Though team composition varied, members often included field veterinarians and ecologists/wildlife biologists for animal sampling; medical doctors, nurses, phlebotomists, or other public health paraprofessionals for human sample collection; and anthropologists, sociologists, community health workers, or other public health professionals for behavioral interviews. Field work and data collection were tightly coordinated, with community engagement, behavioral risk investigations, animal sampling, and human sampling often occurring simultaneously in targeted communities.

## Results

As many PREDICT global virus discoveries originated from bat hosts, analyses across all sites where qualitative data was collected focused on bat interfaces, in addition to large wild animal markets which focused on all taxa in the animal value chain.

**Fig. 4:**
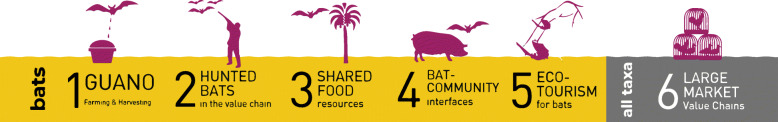
Bat-specific interfaces investigated by One Health surveillance teams. Several bat-specific interfaces were investigated by PREDICT’s One Health surveillance teams and explored in-depth through our human behavioral risk investigations. Large market value chains were a principal area of interest in relation to all wildlife taxa.

Qualitative interviews and focus groups were collected across 13 countries, and were analyzed according to the following 6 risk interface domains, where humans come inregular contact with bats and other taxa (Fig. 4):

### Guano farming and harvesting

In Vietnam, the guano collection process was identified as an important risk interface in agricultural communities, exposing harvesters, vendors, and farmers purchasing guano at risk for transmission of bat viruses. Roles of individuals within the value chain were characterized, confirming guano collection and adjacent domestic animal holdings as a priority for human, bat, and domestic animal surveillance activities.

### Hunted bats in the value chain

Based on food practices in Indonesia, certain occupations are characterized as high-risk: hunters, wild meat processors/slaughterers, vendors, and consumers. Study participants, targeted for their involvement in the wildlife trade, described contact with multiple taxa, with rats, bats, and wild boar being most hunted, transported and sold by the respondents. Most individuals interviewed lacked knowledge of potential zoonotic disease threats, with only a few respondents under the perception that a wild animal can cause sickness. In North Sulawesi, over half (54%) of participants reported having treated a bite or scratch received while slaughtering, and 83% (*n* = 145, CI 0.77–0.88) of those individuals had contact with bats. These preliminary findings indicate that people in high-risk occupations need to be better informed about zoonotic disease transmission.

In Côte d’Ivoire, bat hunters described hunting with a slingshot, a catapult, or a gun, then killing the bat with a final blow of a machete once it fell to the ground. Several participants mentioned specifically handing off the dead bats to children for preparation. When handling the dead bat, respondents described bare-handed contact with the bat body and blood. Some respondents revealed that according to tradition, bat consumption is discouraged among pregnant women. Eating bats was described by some as a custom of the past, others expressing distaste for bats, and a few said bats were appetizing. While some respondents reported bats as rare or difficult to find, others described their regular presence around their homes and fields. Possibly due to previous public health messaging regarding Ebola transmission risk from bats, respondents seemed uneasy discussing bats with the interviewers.

### Shared food resources between bats and humans

In Bangladesh, wild macaques interact with the community, frequently entering kitchens and other domestic areas, opening food and water containers, riffling through clothing, and stealing food. Regular physical contact with the macaques was reported, including bites and scratches. Some attributed these conflicts to a change in macaque home ranges driven by deforestation, where they now must invade houses to find food.

In Tanzania, participants shared insights about wildlife raiding their crops. Destruction of crops by baboons and other non-human primates, rodents, and wild and domestic ungulates is such a severe problem that some farmers spend from several weeks to 7 months living in temporary shelters in their fields to scare animals away. Participants talked about the increasing scarcity of wild meat and attributed this to increased human population density, and described a range of conditions under which people eat the meat of animals known to have died of disease. Another pervasive theme was the claim that refugee populations drive hunting and bushmeat consumption, particularly of non-human primates.

### Bat Community interfaces

In Sierra Leone, participants in focus group discussions and interviews revealed direct and indirect contact with bats, and were not aware of the potential health risks posed from human-bat contact. They shared that insectivorous bats were seen as pests, were commonly found roosting in roofs of homes, and their excreta (urine and feces) were contaminating food and water supplies. While individuals were aware that bats were implicated in the Ebola epidemic, they did not have an adequate understanding of how zoonotic diseases are transmitted or of the health risks that bats pose. Community members recalled hearing public health messages concerning bats and wildlife during the Ebola epidemic, but they were unsure of the messages’ veracity or relevance. The knowledge-focused health interventions implemented during the Ebola outbreak seem to have fallen short of motivating long-term behavior change, as virtually all of the hunters had resumed bat hunting by the resolution of the outbreak.

In Nepal, community-based surveillance in urban Kathmandu and in rural areas engaged participants about contact with wild and domestic animals, which was prevalent in all communities. Bat hunting was most common in male and adult (20–60 years) participants. People who hunted bats were also more likely to report influenza-like symptoms in the past year, compared to individuals who did not hunt bats. The majority of people in rural and some from urban communities reported that they had eaten raw, sick animals, or animals found dead. Hunting of wild animals and selling of dead animals were observed only in the rural study site, where communities engaged in hunting were targeted for surveillance and where residents reported hunting, cooking, or handling bats. Other animals were also hunted in this community to mitigate crop and food raiding. Respondents from rural communities provided important context for their contact with animals, including bats, where findings highlight concerns over sanitation and hygiene, lack of knowledge on disease risks, and the prevalence of high-risk activities for virus spillover related to human-animal interactions. These behaviors and documented knowledge gaps show vulnerabilities to infectious diseases.

### Ecotourism

In Cameroon, tourists visit a mountaintop near the village of Ndem-Mvo’h where they go to pray to what is known to the locals as the “Hidden God” or “God of the Cave.” This sacred cave contains a large colony of thousands of *Rousettus aegyptiacus* bats and is used in rituals in which people come to purify themselves and ask for good fortune and blessings. Though bat hunting is forbidden by the local Chief, hunting bats for sustenance is done in secret, as reported by a local. The ecotourism interface is an important one in this area, as the Cameroonian population has high levels of sustained contact with bats, with most people exercising little if any form of personal protection. Locals stress that it is difficult to restrict activities at the cave due to economic barriers and years of cultural practice.

In the Kongo Central region of the Democratic Republic of Congo, there are multiple tourist attractions bringing people to DRC from all over the world: the Luki Biosphere Reserve (a protected UNESCO site), Zongo Falls Park & Lodge, the Mangroves Marine Park in Moanda, and the Grand Inga Dam, one of the largest hydroelectric power plants in the world. During a focus group in Inga, hunters talked about hunting fruit bats seasonally, from October to December, in huge numbers. Hunters sell to private clients or to the village women, who sell to their wide network of local buyers and tourists in the surrounding cities, including Kinshasa. “We organize ourselves to go hunting on Saturday, and we have [clients’] phone numbers and when we slaughter, we will call them to tell them that now we have such an animal, so you come meet us in such a place and we sell it.” The ecotourism interfaces around the region create the possibility of disease transmission and spread over long distances, with tourists potentially being exposed to zoonotic diseases and returning home with them.

### Large market value chains

In the Republic of Congo (RoC), bats were a visible staple in the markets in Brazzaville. Bats were described as being available for purchase either freshly killed and ready to be butchered at home, or alive and available to be slaughtered on demand in the market. In interviews with wild animal value chain actors -- hunters, suppliers/middlemen, vendors, consumers, and employees of adjacent shops and businesses -- among those who reported slaughtering bats (putting them into direct contact with the viscera), bats were seen as having little that could not be consumed. Bat hunting, particularly during mango season, could yield substantial income for a family, and selling bats was perceived as the only way to make ends meet for some households. When asked about Ebola and the risks associated with bat consumption, an adult female shop owner shared her perception, “Since we saw nobody die of it, we keep eating them.” Other participants said that though they neither sell nor eat bats, they sometimes handle bats in the course of preparing meals for other household members. Smoked bushmeat was also a recurring preference.

In Cameroon market sites, the team recorded sales of nearly 40 different species of wild animals, many of which are protected species that are illegal to hunt or to sell, and several of which are endangered due to their low remaining population sizes in the wild and frequent involvement in illegal trafficking. Prices ranged widely for different species, and the demand for the wild meat was high. Market workers and hunters interviewed said that bushmeat does not transmit illness to people, and that transmission of disease cannot occur between animals and humans. Some market workers and butchers say that working with wild animals is not risky. Many believe that the only risk is of cutting oneself, not due to blood-blood contact between animals and humans, but because the wound may get infected if not treated properly. Most market workers and hunters do not consider PPE important. Several mention that gloves are not a feasible protective measure, as “hospital-style gloves are too thin to protect against anything, and larger gloves used for heavier tasks are too cumbersome for the work we do.” According to a restaurant worker, people in Sangmelima do not hunt or consume bats, as their physical appearance is off-putting to many, with a few individuals explaining that “they are too ugly to eat.”

In DRC, bushmeat vendors expressed a lack of knowledge of disease transmission, particularly about the role of animals in the transmission of illness. Most vendors reported that wild animals cannot carry disease and therefore could not transmit diseases to humans. Bushmeat is perceived to be “natural” since it is not raised by humans. Some said they had heard Ebola was spread by animals but others attribute Ebola to witchcraft. Multiple bushmeat vendors stated, “This story of Ebola is false. There was a trapper whose animal was stolen, and to get revenge he made a fetish and he killed all those who had eaten his meat....[Clients] continue to eat bushmeat because they know that it wasn’t Ebola but rather a history of bushmeat and witchcraft.” Nearly all bushmeat butchers avoid cleaning their butchering utensils or the work surfaces, as they say the taste of the bushmeat would be ruined by the soap. Many butchers wear clothes for market work that are kept separate from home clothes. PPE such as gloves, masks, or boots, were rarely used by bushmeat vendors or butchers.

**Fig. 5:**
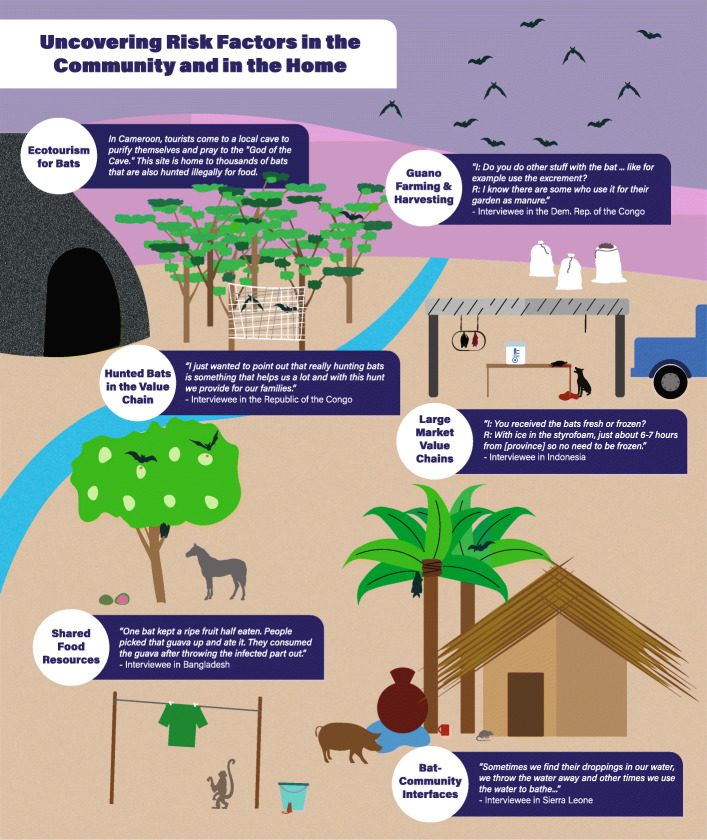
While human-bat interactions were unique by country and context, key themes ranging from bat hunting to bat-community interfaces were commonly shared among the countries conducting qualitative research. This figure presents cross-country examples of how interviewee’s describe local interaction with wildlife, particularly bats

Insights gained through in-depth behavioral investigation provided valuable information for the development of culturally appropriate interventions and behavior change communication. Meetings with government partners were held to raise awareness of zoonotic diseases and to identify potential strategies for risk reduction and disease prevention (Fig. 5).

### Assessing behavioral risk and operationalizing one health surveillance

From an epidemiological perspective, behavioral risk assessments often seek to quantify the influence of known risk factors on disease emergence and transmission dynamics. PREDICT’s focus also aimed to identify and assess a range of known and unknown socio-cultural behaviors that could be influential in zoonotic disease emergence, amplification, and transmission. This broad approach to behavioral characterization enabled us to identify and characterize a milieu of human activities that could be later studied to investigate the transmission dynamics of new and emerging viruses. For diseases for which etiologies are known and characterized, such as zoonotic Influenza infection, this approach allowed us to determine behaviors that might be risk factors for certain groups (e.g., agricultural workers) and to better understand the socio-cultural contexts necessary to develop effective risk mitigation strategies.

**Fig. 6:**
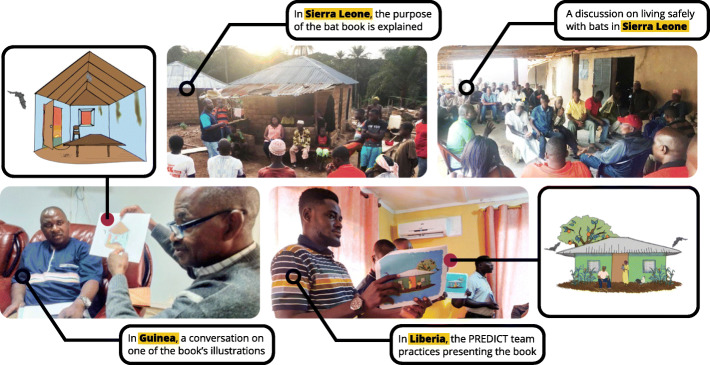
Implementation of the Living Safely with Bats resource in West Africa, which provided scripted talking points for moderators. These talking points covered themes such as basic ways to live safely with bats, disposal of dead bats, what to do with them when contact is unavoidable, and managing bats living in and around the home

Throughout implementation, our teams built partnerships and relationships at the national, subnational, and community levels. Before the roll-out of activities, our staff worked with a range of municipal and traditional stakeholders, including officials, leaders, chiefs, and elders in the target communities, to help One Health teams effectively engage with communities and to facilitate permissions and access for animal and human sampling efforts. Our teams also conducted site scoping visits, and in some cases formative behavioral risk research in collaboration with ministry partners, which helped determine One Health surveillance priorities and at-risk site selection. Through this multi-level stakeholder engagement process, our staff were able to build relationships and teams necessary for gaining community buy-in, trust, and support for our unconventional surveillance strategy (Fig. 6).

Based upon qualitative insights about the geographic origin of bushmeat coming into Kinshasa markets, we traced the animal value chain back to Mbandaka, the reported source of much non-human primate meat. Mbandaka is an Ebola outbreak site, so we used our interview data to generate hypotheses about Ebola exposure through bushmeat butchering, and did further sampling and serology of primates and bush meat vendors to test this hypothesis (Lucas et al., 2020).

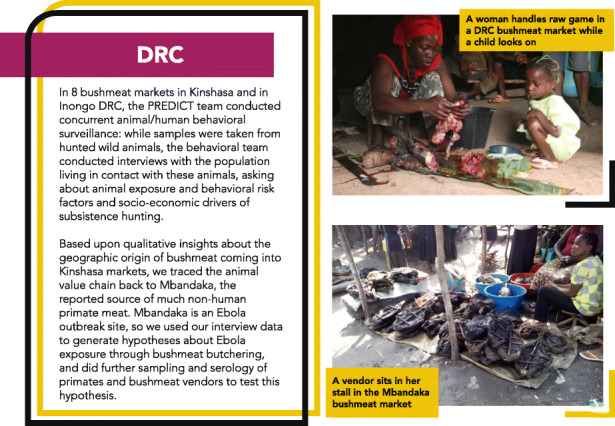



Photo caption: A woman sells wild meat at the Mbandaka market, one of the sources for wild meat entering the terminal urban markets in Kinshasa and a location for previous Ebola outbreaks. (Credit: PREDICT Consortium).

## Discussion

### Insights and impact from incorporating social sciences into one health surveillance

In most countries, the teams charged with implementing the behavioral risk strategy were composed of both new and seasoned scientists from diverse professional backgrounds. Through standardized trainings aimed at strengthening skills and techniques needed in both the behavioral and biological scopes of PREDICT, we helped encourage a collaborative and multidisciplinary surveillance workforce that leveraged the experiences and skills of the broader team. Cross-training staff also enabled and facilitated the close integration and coordination of our behavioral risk strategy with One Health surveillance and sampling efforts, and project scientists were able to investigate the attitudes, beliefs, behaviors, and broader social contexts of targeted at-risk populations. This tight integration allowed our teams to conduct rapid assessments of community risks during early formative research, and eventually to develop truly multidisciplinary behavior change communication and risk reduction plans relevant to communities and stakeholders they engaged. Further, the inclusion of social scientists into animal surveillance teams strengthened zoonotic disease surveillance, as community knowledge and practices acquired through social science research helped inform the timing of wildlife sampling and identify additional locations for sampling and surveillance efforts. Our trained social science teams helped raise awareness about taboos or socio-cultural sensitivities that needed to be considered when developing and refining surveillance plans.

PREDICT’s surveillance approach was designed to balance human health and conservation objectives with wildlife sampling targets. Animal species were live captured and released after sample collection. In communities where rodents are known to cause human illness, such as Lassa fever in the West Africa region, our teams needed to work closely with community members to explain the methods and context of this program, gain buy-in for sampling activities, and help identify effective strategies to minimize rodent contact and exposure. PREDICT sampling teams frequently refrained from engaging in animal sampling until sufficient time was spent with the community to gain their trust, often through dialogue on possible interventions and by providing and presenting specially tailored risk reduction recommendations.

Integrating the social sciences into PREDICT’s One Health surveillance approach provided a range of secondary benefits beyond our primary goals. These included: building support, trust, and buy-in of populations hosting or involved in One Health initiatives; contributing sociological and anthropological insights on human activities to guide geographic targeting of surveillance initiatives; crafting “empathetic approaches” to behavioral interventions – either to mitigate outbreak risk or respond to outbreaks; and designing and implementing One Health interventions among at-risk populations.

In the spirit of community-based participatory research which integrates mutual education (between researchers and community experts) and social action in improving health, our PREDICT teams engaged national and subnational leadership and facilitated meetings with provincial/local authorities, allowing us to directly engage communities in project activities and the research process. In many cases, our country teams returned to communities every 3–6 months to sample and conduct interviews. Through these frequent interactions, teams gained trust with community members, an essential element which helped improve the richness and depth of interview data over time. In addition, towards the end of the project, between May and September 2019, our teams returned to these communities equipped with summaries and reports of available project findings along with risk reduction materials specially tailored to the unique human-animal-environment interfaces investigated by the surveillance teams. Returning to participating communities to share project findings is unfortunately extremely rare. We received reports from nearly all countries that community members were extremely grateful to hear about project findings along with our team’s recommendations for improving health and conservation. Our team strongly recommends that planning and budgeting for community engagement to share findings and recommendations at the end of a project is critical, ethical, and should be part of all project designs.

In Sierra Leone, the PREDICT country team was able to rapidly deploy behavioral researchers to study populations exposed to high-risk bat interfaces in and around sites where the wildlife surveillance team had recently detected a new Ebolavirus species - *Bombali ebolavirus*. The early integration of behavioral research with wildlife surveillance enabled the team to quickly assess potential exposure pathways in order to inform the development of public health communications tailored to the affected populations. Communications included specific messaging for the behaviors, contexts, and interfaces identified in the region, with special emphasis on household bat infestations and bat hunting.

Many of the Sierra Leone behavioral team members were former contact tracers from the West Africa Ebola outbreak. After having conducted over 100 interviews with the PREDICT behavioral research tools, both field interviewers reported that the training they received had greatly improved their interviewing skills, allowing them to obtain more nuanced information and better prepare them for future public health investigations.

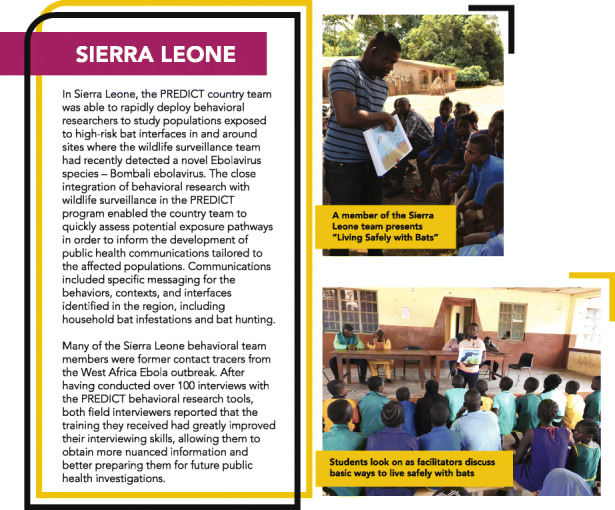



Photo caption: The Sierra Leone team delivers the behavior change communication and risk reduction resource *Living Safely with Bats* to community members during an outreach and risk communication campaign following the discovery of a new Ebolavirus in bats. (Credit: PREDICT Consortium).

### Social science insights on targeted surveillance

During formative research and the selection of surveillance sites, local subject matter experts or ‘guides’ provided entrée into what were often closed, tight-knit communities. Ethnographic interviews allowed for open-ended dialogue about target interfaces and the underlying dynamics and drivers of human activities that, from a public health/disease transmission perspective, could be considered ‘risky’, such as eating bats, rodents, or non-human primates, or drinking raw blood. Some risk behaviors are considered taboo from one community to another, based on tribal, ethnic, or social beliefs, and these differences had to be explored, acknowledged, and respectfully addressed. One important approach was to enroll local interviewers, when possible, or local translators who spoke local dialects, who could clearly explain the purpose of the study and reasons for blood collection (a highly suspect procedure in many cultures), as well as the need to sample their animals (also a barrier for many, as animals, whether domestic or wild, are prized commodities and sampling was sometimes seen as damaging/tainting the meat or reducing its value). Early focus group discussions helped describe practices and beliefs about disease that warranted further exploration, and also catalyzed information exchange between our teams and local experts, which often informed the selection of sites where sampling would take place. For example, through discussions with bat hunters we learned about the location of bat roosts or caves for sampling, and in conversations with bushmeat vendors, we were directed to villages where they bought hunted meat and where we could move further upstream in the bushmeat value chain.

### Designing and implementing one health interventions with at-risk populations

As PREDICT’s laboratories detected and confirmed virus findings, including new discoveries of potentially dangerous pathogens, it became imperative to engage our host country government partners and community stakeholders to share these findings along with recommendations for continued surveillance and risk reduction. In Sierra Leone for example, PREDICT scientists discovered a new ebolavirus in bats, Bombali virus, which was the first time an ebolavirus had been detected in wildlife before causing human infection [7]. The sampling sites for these bats were close to villages and human dwellings, as by design our surveillance sites were selected to explore high-risk areas characterized by increased interactions between animal and human populations. A potentially deadly virus detected in an animal necessarily requires an empathetic and strategic human (public) health response. By using some of the contextual data about human exposure, collected through behavioral risk investigations, our team worked collaboratively with PREDICT Consortium ecology, bat biology, and virology experts to design and develop a rapid intervention strategy.

To identify the most culturally appropriate, feasible, and effective intervention resource format, our team developed a framework for assessing potential materials, channels of communications, respective audiences, and core messaging. A moderated picture book format, delivered by a trusted community leader, was selected as the best tool to put into the hands of our local team and in-country stakeholders. A communications plan was developed to ensure a well-coordinated effort and timely discussions with government and community stakeholders, following the release of the new Bombali virus finding [7]. The resource, entitled *Living Safely with Bats* [18], leveraged the collective subject matter expertise of the consortium and featured illustrations from a team member trained in animal biology and visual arts ensuring accurate, consistent, and compelling visual representations. To refine and test the book format and key messages, focus groups were held with project subject matter experts and feedback was solicited from project country teams. The book’s content benefited from cultural vetting by 17 country teams (Bangladesh, Cambodia, Cameroon, Côte d’Ivoire, DR Congo, Ghana, Guinea, Indonesia, Lao PDR, Malaysia, Nepal, ROC, Senegal, Sierra Leone, Tanzania, Thailand, and Vietnam).

**Fig. 7:**
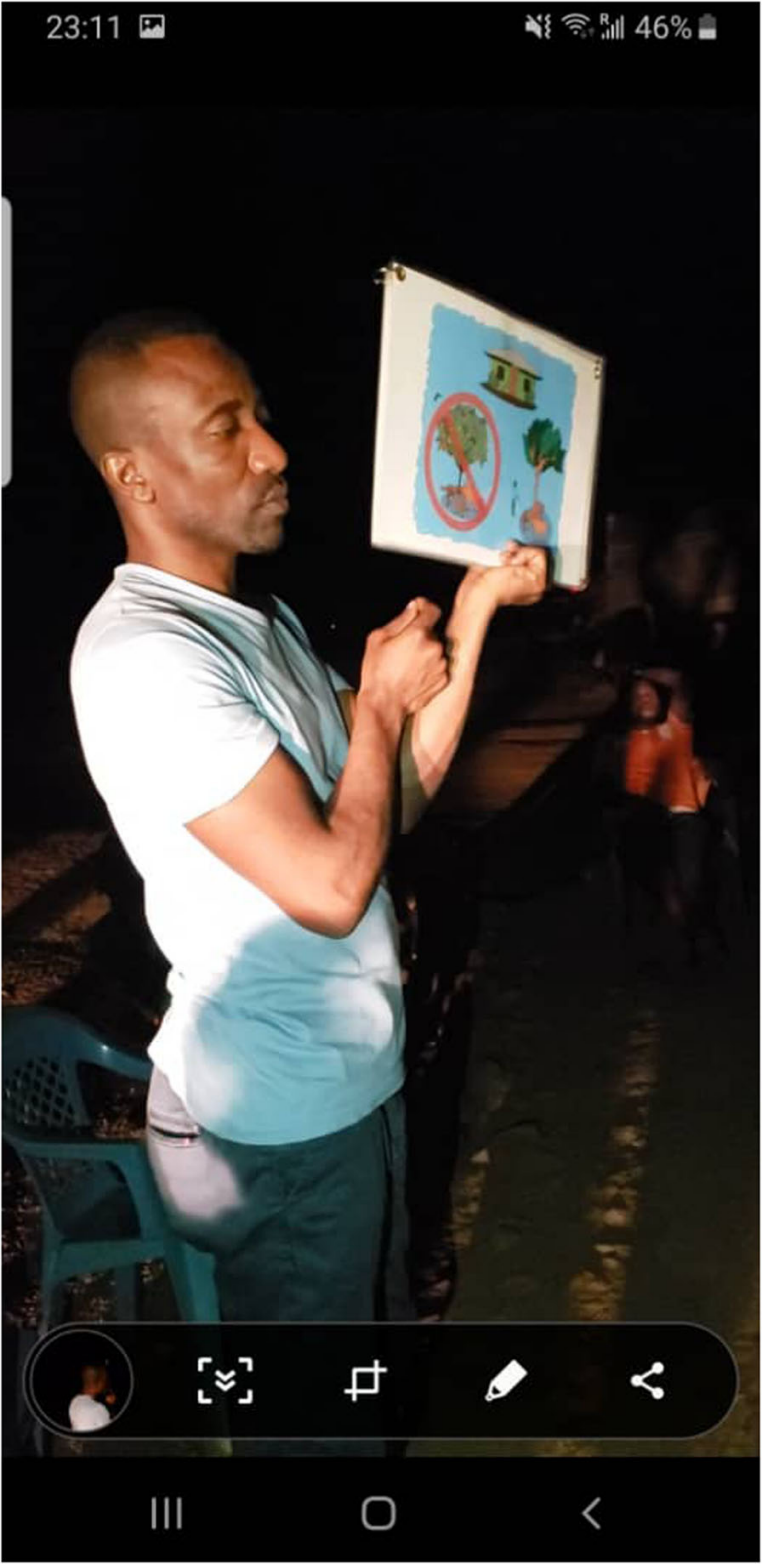
Community Engagement in DRC: Sharing Living Safely with Bats with partner communities: Talking points included themes such as bats as essential agents in the local ecosystem

Consortium experts, including our behavioral risk team embedded with our staff scientists in West Africa, helped train and support the implementation of the *Living Safely with Bats* resource during community outreach events in Sierra Leone, Guinea, and Liberia beginning in July and August 2018. Country teams utilized the resource in a variety of formats: official briefings with ministry partners, in-person presentations and community meetings, classroom sessions in local primary and secondary schools, and local radio broadcasts. In Guinea, radio broadcasts reached thousands of individuals across the entire Forest Region – the area where the 2014 West Africa Ebola epidemic originated, likely via a spillover event from a bat [23]. This resource has been translated into 12 languages, including Amharic, Bahasa, Burmese, Dusun, English, French, Khmer, Kiswahili, Lao, Malay, Thai, and Vietnamese. The book was also adapted to share with the communities that PREDICT teams throughout Asia had engaged and worked with over time. Changes to content in this version included artistic modifications to incorporate locally salient fruits, foliage, and protective clothing items, in addition to content addressing Asia specific human-bat interfaces identified as particularly high-risk for virus spillover (date palm sap collection, bat guano farming and harvesting practices, and cave-related tourism) (Fig. 7).

After the Bombali virus was discovered in bats [7], our teams focused more specifically on bat-human interfaces for virus spillover and transmission. Country teams identified specific bat-human interfaces that might serve as potential zoonotic spillover sites for more targeted sampling, and in countries engaged with in-depth qualitative investigations, ethnographic interviews and focus group discussions were conducted with individuals connected to a range of hypothesized at-risk bat-human interfaces, described above. This approach led to the design of several deep-dive analyses seeking to directly link behavioral data with ecological and biological data to generate exploratory risk models, estimate the potential impact of risk reduction strategies, and ultimately identify candidate intervention recommendations that could be pilot tested and if successful, taken to scale.

### Sharing our findings for sustained one health engagement

Involving at-risk communities in disease hotspot areas is critical, both for developing awareness of disease risk and encouraging community agency to define realistic strategies for disease mitigation for their particular community. During the last year of the project, as our country teams returned to participating villages and communities to share results, community members wanted and encouraged the team to share findings beyond just their local area, as they realized the value of the project’s broader impact, and wanted to share this knowledge with other neighboring at-risk communities. They voiced appreciation of the value of One Health data and evidence, from the wildlife we captured and sampled and the viruses we detected, to the unique animal-human contexts identified as risks for infection. Even though risk reduction or intervention strategies we identified might imply changes in behaviors that have been ongoing for generations (ie. bushmeat hunting), their interest and willingness to engage demonstrates the power of community buy-in that is needed for prevention and sustained disease control efforts.

A major lesson learned towards the end of the project was managing expectations as project activities concluded. Due to the One Health surveillance design, our teams collected a vast quantity of data, an enormous and valuable archive of information that required a tremendous amount of data review for quality assurance prior to use. Taking inventory of this archive of hundreds of thousands of data points – generated from human and animal biological specimen data (including geolocation coordinates, other metadata, and virus level results) and data from behavioral questionnaires and qualitative interview transcripts – was a massive undertaking for Consortium staff at both the global and country levels. Sharing findings with stakeholders involved in the project was an obligation that the team took very seriously, but sharing such findings raises questions about what communities can do to protect themselves from emerging disease threats. To this end, our teams spent time with these at-risk communities, conducting focus groups to discuss what might be attainable, and exploring protective interventions that communities might adopt to prevent zoonotic disease exposure. By working with this data and the knowledge and insight gained by our local teams, we developed intervention recommendations that were then presented back to communities at the conclusion of the project.

Although the primary objectives of PREDICT did not involve developing or testing behavioral risk interventions, we conducted in-depth behavioral risk investigations to better understand the individual- and community-level knowledge, attitudes, and practices that potentially put key actors at risk for zoonotic disease transmission from the animals they live and work with. When we asked highly-exposed individuals (ie. bushmeat hunters, wildlife or guano farmers) about the risk they perceived in their occupational activities, most did not perceive it to be risky, whether because it was normalized by years (or generations) of doing such an activity, or due to lack of information about the potential risks. Many individuals spoke about the occupational-exposure to animal disease risks not being as important as earning a living, so as with Maslow’s hierarchy of needs, physiological needs such as food, water and shelter, usually come before ‘safety needs’. Satisfying those ‘physiological needs’ requires a cash income. Participants almost uniformly referred to the socio-economic drivers of the work they do handling animals, and some spoke about not having other choices.

## Conclusions and recommendations

To be successful, emerging infectious disease surveillance projects cannot focus on surveillance and detection of pathogens alone. Integrating social science into these projects facilitates more comprehensive investigation into the specific human activities that are hypothesized to drive disease emergence, amplification, and transmission, in order to better substantiate behavioral disease drivers, along with the social dimensions of infection and transmission dynamics. Understanding these dynamics is critical to achieving health security -- the protection from threats to health -- which requires investments in both collective vulnerability and individual health security [10]. Collective approaches are often at the policy level and focus on health security at scale and may include strengthening surveillance and detection systems, quarantine efforts, vaccination campaigns, border controls, and real-time reporting of any public health emergency of international concern. Through initiatives such as the Global Health Security Agenda, there are commitments to health system strengthening and policy adherence to International Health Regulations at the national level. Despite these promising developments, individual or community health security is a crucial if nebulous domain of focus, as is the important role that social sciences can play in its realization. In an outbreak situation, individual health security is framed as critical “personal access to safe and effective health services, products, and technologies” (Ibid). The work of our social science teams however, demonstrates that investments in disease prevention upstream through efforts targeting behavior change helps individuals better understand disease transmission dynamics, can change risky behaviors, and can further enhance both individual and community health security.

Effective behavioral change strategies must be evidence-based and begin with education and meaningful messaging that is focused on the target audience’s concerns. These include socio-economic factors as well as factors impacting individual and community health security. Identifying health risks and suggesting potential mitigation strategies can be achieved in a relatively short time, as we have demonstrated through our work at the bat-human interface. However, encouraging uptake and achieving lasting behavioral change praxis in diverse cultures and communities requires committed, prolonged engagement, as well as evaluations to test and explore the impact of interventions. For example, further research is needed to better understand which behaviors individuals will change, if at all, and what effect these changes will have on health outcomes.

Perhaps the most positive outcome from PREDICT has been the integration of social science approaches with One Health surveillance to work towards community-level resiliency. Our global Consortium worked for over 10 years to strengthen the capacity for local scientists to safely, ethically, and humanely put One Health in action from the identification of at-risk communities and sites for wildlife and human sampling activities to collection and testing of those samples, and finally for sharing findings with our global, national, and local stakeholders. Ultimately, however, it was the involvement of behavioral sciences that allowed us to push toward fuller community integration and engagement and toward dialogue and implementation of recommendations for disease prevention and improved health security. However, the adoption of these recommendations and ultimately the sustained engagement required to evaluate their success remains a high priority for further investment.

We encourage future programs to work with communities on educational and capacity-building initiatives that improve community awareness of disease threats and that work collaboratively towards risk mitigation strategies. Additionally, we encourage further integration of social sciences in disease surveillance programs to better identify and illuminate specific and previously unidentified drivers of disease emergence and spread for individual pathogens, especially between natural and social systems. While our strategy was designed for identifying and comparing risks for human and animal contact and potential exposure to a diversity of zoonoses across countries, the broader socio-cultural and economic dimensions of risk that emerged from our ethnographic in-depth behavioral investigations warrant further exploration, especially with regard to specific wildlife-human interfaces, the associated behaviors and practices that might influence virus spillover and spread, and the potential suite of targeted interventions that could be explored for effective risk reduction and disease control.

As we enter a new decade, the One Health approach continues to break down barriers between silos in the scientific, health, and security communities and importantly bridges divides between natural and social systems. For the latter, PREDICT provides a new model and framework for transforming theory into practice, for “socializing” One Health and beginning to take it to scale.

## Supplementary Information

**Additional file 1.** Human questionnaire administered by 24 countries as part of the human surveillance scope.

**Additional file 2.** Ebola Host Project questionnaire administered in Guinea, Liberia, and Sierra Leone.

**Additional file 3.** Ethnographic interview and focus group discussion guides administered in 13 countries during in-depth qualitative behavioral risk investigations.

## Data Availability

The questionnaires, ethnographic interview and focus group discussion guides are attached with this manuscript submission. PREDICT standard operating procedures, guides, and select training materials used for the implementation of this study are available at: https://ohi.vetmed.ucdavis.edu/programs-projects/predict-project/publications#Guides
