# Supplementary material for: Socializing One Health: an innovative strategy to investigate social and behavioral risks of emerging viral threats
Source: One Health Outlook. 2021 May 14;3:11. doi: 10.1186/s42522-021-00036-9 (PMC8122533; doi:10.1186/s42522-021-00036-9)
Supplement: Supplementary file 1 — Additional file 1. Human questionnaire administered by 24 countries as part of the human surveillance scope. [file 42522_2021_36_MOESM1_ESM.zip › Socializing One Health Surveys/DwellingsR1.pdf]

Dwellings, Buildings and Temporary Settlements Module

V 1.3

Add Site and Event Form ID:

Site name and date:

____

(For reference only)

0	1	2	3	4	5	6	7	8	9
0	1	2	3	4	5	6	7	8	9
0	1	2	3	4	5	6	7	8	9
0	1	2	3	4	5	6	7	8	9
0	1	2	3	4	5	6	7	8	9
0	1	2	3	4	5	6	7	8	9

1. Is this permanent or temporary human settlement? Select all that apply.
   - ○ permanent
   - ○ temporary

2. What is the type of dwelling at this sampling event site? Select all that apply.
   - ○ home
   - ○ work
   - ○ congregation sites
   - ○ schools/universities
   - ○ abandoned building

3. Which animal taxa have you observed inside the dwelling and why? Select all that apply for each row.

	not present in dwelling	unwanted animals entering structure on their own	animals near food preparation and eating areas	animals kept as pets	animals raised or kept for food	butchering of animals on site
rodents/shrews	○	○	○	○	○	○
bats	○	○	○	○	○	○
non-human primates	○	○	○	○	○	○
birds	○	○	○	○	○	○
carnivores	○	○	○	○	○	○
ungulates	○	○	○	○	○	○
pangolins	○	○	○	○	○	○
poultry/other fowl	○	○	○	○	○	○
goats/sheep	○	○	○	○	○	○
camels	○	○	○	○	○	○
swine	○	○	○	○	○	○
cattle/buffalo	○	○	○	○	○	○
dogs	○	○	○	○	○	○
cats	○	○	○	○	○	○
unknown taxa	○	○	○	○	○	○

4. Is there a healthcare facility that can be accessed within 1 day?
   - ○ yes
   - ○ no

5. If yes above, do they have:

	yes	no
capacity for medical treatment?	○	○
capacity for preventive services?	○	○
a posted ebola or other disease outbreak preparedness plan?	○	○
capacity for pharmacy services?	○	○

example only do not use in field

231011-930186
