# Supplementary material for: Socializing One Health: an innovative strategy to investigate social and behavioral risks of emerging viral threats
Source: One Health Outlook. 2021 May 14;3:11. doi: 10.1186/s42522-021-00036-9 (PMC8122533; doi:10.1186/s42522-021-00036-9)
Supplement: Supplementary file 1 — Additional file 1. Human questionnaire administered by 24 countries as part of the human surveillance scope. [file 42522_2021_36_MOESM1_ESM.zip › Socializing One Health Surveys/MarketValueChainR1.pdf]

Market and Value Chain Module

EIDITH
V 1.2

Add Site and Event Form ID:

0	1	2	3	4	5	6	7	8	9
0	1	2	3	4	5	6	7	8	9
0	1	2	3	4	5	6	7	8	9
0	1	2	3	4	5	6	7	8	9
0	1	2	3	4	5	6	7	8	9
0	1	2	3	4	5	6	7	8	9

Site name and date: ____________________

(For reference only)

1. What is the node along value chain? Select one option.
   - source of animals (area of capture or farm)
   - animals in transit
   - distributer/middle man
   - point of sale

2. How many vendors or workers are at the event site? Select one option.
   - only 1 person
   - <5 people
   - 5-20 people
   - 21-100 people
   - >100 people

3. Which animals are for sale in the market or value chain at this event site? Select all that apply for each row.

	live	dead	parts	slaughtered at site	none observed
rodents/shrews					
bats					
non-human primates					
birds					
carnivores					
ungulates					
pangolins					
poultry/other fowl					
goats/sheep					
camels					
swine					
cattle/buffalo					
dogs					
cats					

4. What is the maximum number of taxonomic groups in one holding area or cage? Select one option.
   - 1
   - 2-5
   - 6-10
   - >10

5. Are wild animals and domesticated animals held together in one holding area or cage?
   - yes
   - no

6. What types of biosecurity measures are practiced at the facility? Select all that apply.
   - hand washing facilities
   - gloves for personnel handling animals or raw animal products
   - protective clothing and footwear for personnel performing butchering and slaughtering
   - washing and disinfecting animal crates and equipment in contact with animals or animal products
   - removal of sick or dead animals from live animal settings
   - no biosecurity observed

7. What type of animal waste is present at the event site? Select all that apply.
   - feces
   - soiled bedding (urine and feces)
   - animal tissue and/or blood
   - none

example only do not use in field

225437-675259
