# Supplementary material for: Socializing One Health: an innovative strategy to investigate social and behavioral risks of emerging viral threats
Source: One Health Outlook. 2021 May 14;3:11. doi: 10.1186/s42522-021-00036-9 (PMC8122533; doi:10.1186/s42522-021-00036-9)
Supplement: Supplementary file 1 — Additional file 1. Human questionnaire administered by 24 countries as part of the human surveillance scope. [file 42522_2021_36_MOESM1_ESM.zip › Socializing One Health Surveys/CropProductionR1.pdf]

Crop Production Module

EIDITH
V 1.3

Add Site and Event Form ID:

0	1	2	3	4	5	6	7	8	9
0	1	2	3	4	5	6	7	8	9
0	1	2	3	4	5	6	7	8	9
0	1	2	3	4	5	6	7	8	9
0	1	2	3	4	5	6	7	8	9
0	1	2	3	4	5	6	7	8	9

Site name and date:

____

(For reference only)

1. Which crops are planted on the site?
   Select all that apply.

- ◯ coffee/tea/cocoa plants
- ◯ fruit/nut trees
- ◯ oil tree plantation
- ◯ oil seed crops
- ◯ dry grains
- ◯ sugar
- ◯ vegetable/fruit crops
- ◯ pulses/legume
- ◯ fiber
- ◯ forages
- ◯ cover crops
- ◯ fallow fields
- ◯ rubber

2. What type of crop production system is it?
   Select one option.

- ◯ family owned
- ◯ corporate
- ◯ government

3. What is the purpose of growing crops on site?
   Select one option for each crop that exists at the site.

	individual household	village level	large scale production	no crop
coffee/tea/cocoa plants	◯	◯	◯	◯
fruit/nut trees	◯	◯	◯	◯
oil tree plantation	◯	◯	◯	◯
oil seed crops	◯	◯	◯	◯
dry grains	◯	◯	◯	◯
sugar	◯	◯	◯	◯
vegetable/fruit crops	◯	◯	◯	◯
pulses/legume	◯	◯	◯	◯
fiber	◯	◯	◯	◯
forages	◯	◯	◯	◯
cover crops	◯	◯	◯	◯
fallow fields	◯	◯	◯	◯
rubber	◯	◯	◯	◯

4. What are the observed practices or signs of practices used for preventing crop raiding and pest damage?
   Select all that apply.

- ◯ barriers around fields
- ◯ barriers on individual trees
- ◯ fire
- ◯ poison
- ◯ traps
- ◯ shooting
- ◯ loud sounds
- ◯ domestic/guardian animals
- ◯ flooding
- ◯ chasing animals out
- ◯ none

5. What types of fertilizers are used on the crops? Select all that apply.

- ◯ chemical fertilizer
- ◯ animal manure or guano
- ◯ none

6. What is the source of manure fertilizer? Select all that apply.

	composted or dried manure	fresh manure	none
poultry/other fowl	◯	◯	◯
camels	◯	◯	◯
swine	◯	◯	◯
cattle/buffalo	◯	◯	◯
bat guano	◯	◯	◯
bird guano	◯	◯	◯

example only do not use in field

250230-730674

7. Are crops stored at this site after harvest?
- ⬭ yes
- ⬭ no
- ⬭ unknown

8. If yes above, what is the storage location of the harvested crops?
Select all that apply for each crop that exists at the site.

	inside dwellings	storage sacks outside	improved silo granary storage	not observed	other (fill in box)	
coffee/tea/cocoa plants	⬭	⬭	⬭	⬭	⬭	
fruit/nut trees	⬭	⬭	⬭	⬭	⬭	
oil tree plantation	⬭	⬭	⬭	⬭	⬭	
oil seed crops	⬭	⬭	⬭	⬭	⬭	
dry grains	⬭	⬭	⬭	⬭	⬭	
sugar	⬭	⬭	⬭	⬭	⬭	
vegetable/fruit crops	⬭	⬭	⬭	⬭	⬭	
pulses/legume	⬭	⬭	⬭	⬭	⬭	
fiber	⬭	⬭	⬭	⬭	⬭	
forages	⬭	⬭	⬭	⬭	⬭	
rubber	⬭	⬭	⬭	⬭	⬭	

9. Is there evidence (direct observation, signs, scat, tracks) of animals feeding on or visiting crop fields or stored crops?
Select all that apply.

	in crop fields	in stored crops	none
rodents/shrews	⬭	⬭	⬭
bats	⬭	⬭	⬭
non-human primates	⬭	⬭	⬭
birds	⬭	⬭	⬭
carnivores	⬭	⬭	⬭
ungulates	⬭	⬭	⬭
pangolins	⬭	⬭	⬭
poultry/other fowl	⬭	⬭	⬭
goats/sheep	⬭	⬭	⬭
camels	⬭	⬭	⬭
swine	⬭	⬭	⬭
cattle/buffalo	⬭	⬭	⬭
dogs	⬭	⬭	⬭
cats	⬭	⬭	⬭

example only do not use in field
