# Supplementary material for: Socializing One Health: an innovative strategy to investigate social and behavioral risks of emerging viral threats
Source: One Health Outlook. 2021 May 14;3:11. doi: 10.1186/s42522-021-00036-9 (PMC8122533; doi:10.1186/s42522-021-00036-9)
Supplement: Supplementary file 1 — Additional file 1. Human questionnaire administered by 24 countries as part of the human surveillance scope. [file 42522_2021_36_MOESM1_ESM.zip › Socializing One Health Surveys/ZoosSanctuariesR1.pdf]

EIDITH
V 1.4

Add Site and Event Form ID:

Site name and date:

____

(For reference only)

0	1	2	3	4	5	6	7	8	9
0	1	2	3	4	5	6	7	8	9
0	1	2	3	4	5	6	7	8	9
0	1	2	3	4	5	6	7	8	9
0	1	2	3	4	5	6	7	8	9
0	1	2	3	4	5	6	7	8	9

1. What is the purpose of holding the animals? Select all that apply.
   - public viewing or education
   - rehabilitation (for release)
   - sanctuary

2. What is the source of these animals? Select all that apply.
   - acquired from pet trade
   - acquired from wildlife market
   - resident
   - acquired from another zoo/sanctuary
   - unknown

3. How many visitors are observed during this event? Select one option.
   - none (local staff only)
   - 1-10
   - 11-100
   - 101-1000
   - >1000

4. What is the origin of these visitors? Select all that apply.
   - local visitors
   - regional visitors
   - foreign visitors

5. What is the number and density of <u>held animals</u> at this facility? Select one option per row. If the answer is NOT "none", then write in the number for each enclosure question.

	none	1-10	11-100	101-1000	> 1000	number of enclosures	average number of animals per enclosure	average size of enclosure (square meters)	average number of freeranging or tethered animals
rodents/shrews									
bats									
non-human primates									
birds									
carnivores									
ungulates									
pangolins									
poultry/other fowl									
goats/sheep									
camels									
swine									
cattle/buffalo									
dogs									
cats									

example only do not use in field

122625-950023

6. Are there multiple taxonomic groups in one holding area or cage?
   - ◯ yes
   - ◯ no

7. Are wild animals and domesticated animals held together in one holding area or cage?
   - ◯ yes
   - ◯ no

8. What type of animal waste is present at the site/event? Select all that apply.
   - ◯ feces
   - ◯ soiled bedding (urine and feces)
   - ◯ animal tissue and/or blood
   - ◯ none

9. What types of biosecurity measures are practiced at the facility? Select all that apply.
   - ◯ hand washing facilities
   - ◯ showering facilities
   - ◯ footbaths
   - ◯ gloves for personnel
   - ◯ protective clothing and footwear for personnel and other visitors
   - ◯ washing and disinfecting crates or other equipment entering the facility
   - ◯ allowing only essential personnel to enter animal buildings
   - ◯ quarantine of new and/or diseased animals
   - ◯ removal and disposal of dead animals
   - ◯ no biosecurity observed

example only do not use in field

122626-950023
