# Supplementary material for: Socializing One Health: an innovative strategy to investigate social and behavioral risks of emerging viral threats
Source: One Health Outlook. 2021 May 14;3:11. doi: 10.1186/s42522-021-00036-9 (PMC8122533; doi:10.1186/s42522-021-00036-9)
Supplement: Supplementary file 1 — Additional file 1. Human questionnaire administered by 24 countries as part of the human surveillance scope. [file 42522_2021_36_MOESM1_ESM.zip › Socializing One Health Surveys/AnimalProductionR1.pdf]

Animal Production Module

EIDITH
V 1.3

Add Site and Event Form ID:

Site name and date:

(For reference only)

0	1	2	3	4	5	6	7	8	9
0	1	2	3	4	5	6	7	8	9
0	1	2	3	4	5	6	7	8	9
0	1	2	3	4	5	6	7	8	9
0	1	2	3	4	5	6	7	8	9
0	1	2	3	4	5	6	7	8	9

1. What is the type of production system? Select one option.
   - family owned
   - corporate
   - government

2. What is the number and density of animals IN PRODUCTION at this facility? Select one option per row. If the answer in the number section is NOT "none", then write in the number for each enclosure question.

	none	1-10	11-100	101-1000	> 1000	number of enclosures	average number of animals per enclosure	average size of enclosure (square meters)	average number of freeranging or tethered animals
rodents/shrews									
bats									
non-human primates									
birds									
carnivores									
ungulates									
poultry/other fowl									
goats/sheep									
camels									
swine									
cattle/buffalo									
dogs									
cats									

example only do not use in field

230603-185725

3. For each taxonomic group, what is the purpose of animal production? Select all that apply for each row.

	for food	for resale of animals	for sale of animal products	for breeding	not observed
rodents/shrews					
bats					
non-human primates					
birds					
carnivores					
ungulates					
pangolins					
poultry/other fowl					
goats/sheep					
camels					
swine					
cattle/buffalo					
dogs					
cats					

4. Are there multiple taxa in one holding area or cage? ◯ yes ◯ no

5. Are wild animals and domesticated animals held together in one holding area or cage? ◯ yes ◯ no

6. For each taxonomic group IN PRODUCTION, what is the type of containment for the enclosure/housing?
Select all that apply for each row.

	free-range (open range)	tethered grazing outdoor	fenced outdoor enclosure	outdoor cage	indoor enclosure in fully enclosed facility	not observed
rodents/shrews						
bats						
non-human primates						
birds						
carnivores						
ungulates						
pangolins						
poultry/other fowl						
goats/sheep						
camels						
swine						
cattle/buffalo						
dogs						
cats						

example only do not use in field

230604-185725

7. Are animals slaughtered and butchered on-site?
- ◯ yes
- ◯ no

8. If yes above, is there a designated area for slaughtering and butchering?
- ◯ yes
- ◯ no

9. What type of animal waste is present at this event site? Select all that apply.
- ◯ feces
- ◯ soiled bedding (urine and feces)
- ◯ animal tissue and/or blood
- ◯ none

10. What types of biosecurity measures are practiced at the facility? Select all that apply.
- ◯ hand washing facilities
- ◯ showering facilities
- ◯ footbaths
- ◯ gloves for personnel
- ◯ protective clothing and footwear for personnel and other visitors
- ◯ washing and disinfecting crates or other equipment entering the facility
- ◯ allowing only essential personnel to enter animal buildings
- ◯ quarantine of new and/or diseased animals
- ◯ removal and disposal of dead animals
- ◯ no biosecurity observed

11. Are there animals living in the human dwellings?
- ◯ yes
- ◯ no

example only do not use in field
