# Supplementary material for: Socializing One Health: an innovative strategy to investigate social and behavioral risks of emerging viral threats
Source: One Health Outlook. 2021 May 14;3:11. doi: 10.1186/s42522-021-00036-9 (PMC8122533; doi:10.1186/s42522-021-00036-9)
Supplement: Supplementary file 1 — Additional file 1. Human questionnaire administered by 24 countries as part of the human surveillance scope. [file 42522_2021_36_MOESM1_ESM.zip › Socializing One Health Surveys/HumanHealthProfessionalR1.pdf]

Human Hospital & Clinic Module for Health Professionals

EIDITH
V 1.0

0	1	2	3	4	5	6	7	8	9
0	1	2	3	4	5	6	7	8	9
0	1	2	3	4	5	6	7	8	9
0	1	2	3	4	5	6	7	8	9
0	1	2	3	4	5	6	7	8	9
0	1	2	3	4	5	6	7	8	9

Add Human Questionnaire Form ID

Participant ID ______________________
(For reference only)

1. What are your human health activities?
   - ◯ hospital or clinic administrator
   - ◯ hospital or clinic custodial worker
   - ◯ hospital or clinic clinician or nurse (medicine specialty)
   - ◯ hospital or clinic clinician or nurse (surgery specialty)
   - ◯ mobile clinic
   - ◯ traditional healer
   - ◯ dispensary or pharmacy

2. Do you have special protective equipment (Example: shoes, masks, gloves) only worn at work?
   - ◯ yes
   - ◯ no

3. If yes, which protective equipment?
   Select all that apply.
   - ◯ shoes/boots
   - ◯ mask
   - ◯ clothes
   - ◯ gloves
   - ◯ gown/apron

4. Is protective equipment used every time you examine or collect specimens from a patient?
   - ◯ yes
   - ◯ no

5. Is the protective equipment cleaned, sterilized or discarded after each use?
   - ◯ yes
   - ◯ no

6. Do you always use disinfectants to clean equipment and hospital areas?
   - ◯ yes
   - ◯ no

example only do not use in field

240591-485426
