# Supplementary material for: Socializing One Health: an innovative strategy to investigate social and behavioral risks of emerging viral threats
Source: One Health Outlook. 2021 May 14;3:11. doi: 10.1186/s42522-021-00036-9 (PMC8122533; doi:10.1186/s42522-021-00036-9)
Supplement: Supplementary file 1 — Additional file 1. Human questionnaire administered by 24 countries as part of the human surveillance scope. [file 42522_2021_36_MOESM1_ESM.zip › Socializing One Health Surveys/HumanExtractiveIndustryR1.pdf]

Extractive Industry Module

EIDITH
V 1.1

0 1 2 3 4 5 6 7 8 9
0 1 2 3 4 5 6 7 8 9
0 1 2 3 4 5 6 7 8 9
0 1 2 3 4 5 6 7 8 9
0 1 2 3 4 5 6 7 8 9
0 1 2 3 4 5 6 7 8 9

Add Human Questionnaire Form ID

Participant ID ____________________
(For reference only)

1. What type of work or industry is conducted here?
   Select one option.
   - ○ underground mining (by shafts or tunnels)
   - ○ open surface mining
   - ○ hydraulic mining (high pressure water)
   - ○ gathering, panning, or collecting
   - ○ oil well/gas field
   - ○ logging
   - ○ other: ____________________

2. What product(s) are extracted?
   Select one option.
   - ○ coal
   - ○ coltan
   - ○ diamond or other gemstone
   - ○ tin
   - ○ gold/silver
   - ○ lead
   - ○ oil/gas
   - ○ timber/plant
   - ○ electricity
   - ○ other: ____________

3. Do you live on the work site?
   - ○ yes
   - ○ no

4. To the best of your knowledge, how many people work at this site?
   Select one option.
   - ○ <10
   - ○ 10-100
   - ○ 101-1000
   - ○ 1001-10,000
   - ○ >10,000

5. How long have you worked at this site?
   Select one option.
   - ○ <1 month
   - ○ 1 month - 1 year
   - ○ >1 year - 5 years
   - ○ >5 years

6. Is there on-site food production?
   - ○ yes
   - ○ no

7. If yes, who pays for the cost to grow the food crops?
   - ○ the company
   - ○ the workers

8. Is there meat available for consumption?
   - ○ yes
   - ○ no

example only do not use in field

104411-811943

9. If yes, where does the meat come from?
   Select all that apply.
   - farmed onsite
   - farmed and purchased from nearby local communities
   - purchased from wholesale market
   - locally caught/hunted
   - bought frozen
   - don't know

10. Is it possible to consume bushmeat/wild animal meat on or near the site?
    - yes
    - no

11. Is there a designated area for rubbish, including animal waste from slaughter/butcher and animal excrement?
    - yes
    - no

12. If yes, do people use the designated location for rubbish?
    - yes
    - no

13. Do any animals raid food supplies or destroy crops?
    - yes
    - no

14. If yes, which animals?
    Select all that apply.
    - rodents/shrews
    - bats
    - non-human primates
    - birds
    - carnivores
    - ungulates
    - pangolins
    - poultry/other fowl
    - goats/sheep
    - camels
    - swine
    - cattle/buffalo
    - dogs
    - cats

15. What is done to stop animals from raiding or destroying food supplies?
    Select all that apply.
    - barriers around fields
    - barriers on individual trees
    - fire
    - poison
    - traps
    - shooting
    - loud sounds
    - domestic/guardian animals
    - flooding
    - chasing animals out
    - nothing

example only, do not use in field
