# Supplementary material for: Socializing One Health: an innovative strategy to investigate social and behavioral risks of emerging viral threats
Source: One Health Outlook. 2021 May 14;3:11. doi: 10.1186/s42522-021-00036-9 (PMC8122533; doi:10.1186/s42522-021-00036-9)
Supplement: Supplementary file 1 — Additional file 1. Human questionnaire administered by 24 countries as part of the human surveillance scope. [file 42522_2021_36_MOESM1_ESM.zip › Socializing One Health Surveys/HumanZoosSanctuariesR1.pdf]

Zoos & Sanctuaries Module

EIDITH
v1.1

0	1	2	3	4	5	6	7	8	9
0	1	2	3	4	5	6	7	8	9
0	1	2	3	4	5	6	7	8	9
0	1	2	3	4	5	6	7	8	9
0	1	2	3	4	5	6	7	8	9
0	1	2	3	4	5	6	7	8	9

Add Human Questionnaire Form ID

Participant ID ____________________
(For reference only)

1. Do you live on site?
   - yes
   - no

2. To the best of your knowledge, how many people work at this site? Select one option.
   - <10
   - 11-50
   - 51-100
   - 101-1000
   - >1001

3. How long have you worked here? Select one option.
   - <1 month
   - 1 month - 1 year
   - >1 year - 5 years
   - >5 years

4. What species do you work with? Select all that apply.
   - rodents/shrews
   - bats
   - non-human primates
   - birds
   - carnivores
   - ungulates
   - pangolins
   - poultry/other fowl
   - goats/sheep
   - camels
   - swine
   - cattle/buffalo
   - dogs
   - cats
   - none   If "none" selected, skip to question 8

example only do not use in field

5. Do you have special protective equipment (Example: shoes, masks, gloves) only worn at work?
   - yes
   - no

6. If yes, which protective equipment? Select all that apply.
   - shoes/boots
   - mask
   - clothes
   - gloves
   - gown/apron

7. When do you use protective equipment? Select all that apply.
   - handling animals
   - slaughter
   - butcher
   - always on at work
   - other: ____________________

293994-692450

Participant ID

(For reference only)

8. Do you always use disinfectant to clean?
   - yes
   - no

9. If yes, do you always use disinfectants to clean the following:
   Select all that apply.
   - animal enclosures
   - food bins
   - counter tops
   - slaughtering/butchering equipment
   - hands
   - special protective equipment
   - floors

10. How often are the animal enclosures cleaned?
    Select one option.
    - daily
    - weekly
    - monthly
    - as needed
    - never

11. Is there a designated area for the disposal of animal waste?
    - yes
    - no

12. If yes, do people use the dedicated area for animal waste?
    - yes
    - no

13. Is there a quarantine period for new animals?
    - yes
    - no

14. Is there a preventative medicine program for the animals?
    - yes
    - no

15. Do any animals raid food or destroy supplies?
    - yes
    - no

16. If yes, which animals?
    Select all that apply.
    - rodents/shrews
    - bats
    - non-human primates
    - birds
    - carnivores
    - ungulates
    - pangolins
    - poultry/other fowl
    - goats/sheep
    - camels
    - swine
    - cattle/buffalo
    - dogs
    - cats

17. What is done to stop animals from raiding or destroying food supplies?
    Select all that apply.
    - barriers around fields
    - barriers on individual trees
    - fire
    - poison
    - traps
    - shooting
    - loud sounds
    - domestic/guardian animals
    - flooding
    - chasing animals out
    - nothing

example only do not use in field

293995-692450
