# Supplementary material for: Socializing One Health: an innovative strategy to investigate social and behavioral risks of emerging viral threats
Source: One Health Outlook. 2021 May 14;3:11. doi: 10.1186/s42522-021-00036-9 (PMC8122533; doi:10.1186/s42522-021-00036-9)
Supplement: Supplementary file 1 — Additional file 1. Human questionnaire administered by 24 countries as part of the human surveillance scope. [file 42522_2021_36_MOESM1_ESM.zip › Socializing One Health Surveys/MainSiteandEventCharacterizationFormR1.pdf]

Site and Event Characterization Form

EIDITH
V 1.4

Site Section

1. Site Name ________________________________

2. Country ____________________ 3. State/Province ____________________

4. District ____________________

5. Site Latitude ____________________ Site Longitude ____________________
Preferred method - decimal degress

Event Section

6. Recorder ____________________

7. Date of Event ____________________
(eg. 2015-Apr-01)

8. Duration of Event (in days)
1 2 3 4 5 6 7 8 9 10
11 12 13 14 15 16 17 18 19 20

9. Where are you sampling within the site for this event?

Latitude ____________________ Longitude ____________________
Preferred method - decimal degress

10. Which one of these 4 options best describes the human density and impact at this event site? Select one option.

- urban
- peri-urban
- rural
- low disturbance

---

11. What is the target animal-human disease transmission interface for this event? *
Select all that apply.

- animal production
- crop production
- dwellings and temporary settlements
- extractive industry
- market and value chain
- natural area
- wildlife restaurant
- zoos and sanctuaries
- human hospital
- outbreak investigation

12. At what interface(s) did sampling occur for...
Select all that apply. *

	animals	humans	none
animal production			
crop production			
dwellings and temporary settlements			
extractive industry			
market and value chain			
natural area			
wildlife restaurant			
zoos and sanctuaries			
human hospital			
outbreak investigation			

* Be sure to download modules for all selected

13. Does a veterinarian care for animals at this event site?

- yes
- no

160834-201514

14. To the best of your knowledge, what is the estimated size of the area for this event in square meters? Select one option.

○ small <650 sq. m.
○ medium 650 - 3500 sq. m.
○ large 3500 – 7000 sq. m.
○ extra large >7000 sq. m.

15. To the best of your knowledge, what is the estimated number of humans and animals (live or dead) within 0.5 km in any direction? Select one option per row.

	none present	1-10	11-100	101-1000	>1000
humans	○	○	○	○	○
rodents/shrews	○	○	○	○	○
bats	○	○	○	○	○
non-human primates	○	○	○	○	○
birds	○	○	○	○	○
carnivores	○	○	○	○	○
ungulates	○	○	○	○	○
poultry/other fowl	○	○	○	○	○
goats/sheep	○	○	○	○	○
camels	○	○	○	○	○
swine	○	○	○	○	○
cattle/buffalo	○	○	○	○	○
dogs	○	○	○	○	○
cats	○	○	○	○	○

16. Is water shared between humans and animals for…
Select one option per row.

	yes	no	unknown
drinking	○	○	○
bathing/cleaning	○	○	○

17. Are the toilets, latrines or other public facilities available for people at this event site...
Select all that apply.

○ well maintained
○ not maintained
○ used
○ not used
○ none observed

18. Where do people at this site get their drinking water? Select all that apply.

○ fully unprotected: pond, uncovered well
○ rainwater harvesting, water trucking
○ fully protected: water network with taps, covered well
○ none observed

19. How long is the average trip to the drinking water sources? Select one option.

○ <5 minutes walking
○ 5-30 minutes walking
○ >30 minutes walking

20. What types of insect vectors are observed at this event site?
Select all that apply.

○ mosquito
○ sand fly
○ tsetse fly
○ tick
○ none observed
○ other: ____________________

21. Are vector control measures used at this event site?

○ yes
○ no

160835-201514
