# Supplementary material for: Socializing One Health: an innovative strategy to investigate social and behavioral risks of emerging viral threats
Source: One Health Outlook. 2021 May 14;3:11. doi: 10.1186/s42522-021-00036-9 (PMC8122533; doi:10.1186/s42522-021-00036-9)
Supplement: Supplementary file 1 — Additional file 1. Human questionnaire administered by 24 countries as part of the human surveillance scope. [file 42522_2021_36_MOESM1_ESM.zip › Socializing One Health Surveys/HumanAnimalProductionR1.pdf]

Animal Production or Abattoir Module

EIDITH
v1.3

Add Human Questionnaire Form ID

0	1	2	3	4	5	6	7	8	9
0	1	2	3	4	5	6	7	8	9
0	1	2	3	4	5	6	7	8	9
0	1	2	3	4	5	6	7	8	9
0	1	2	3	4	5	6	7	8	9
0	1	2	3	4	5	6	7	8	9

Participant ID ____________

(For reference only)

1. Do you live on site?
   - ○ yes
   - ○ no

2. To the best of your knowledge, how many people work at this site? Select one option.
   - ○ <10
   - ○ 10 - 100
   - ○ 101 - 1000
   - ○ 1001 - 10,000
   - ○ >10,000

3. How long have you worked here? Select one option.
   - ○ <1 month
   - ○ 1 month - 1 year
   - ○ >1 year - 5 years
   - ○ >5 years

4. Which animals are raised here? Select all that apply.
   - ○ rodents/shrews
   - ○ bats
   - ○ non-human primates
   - ○ birds
   - ○ carnivores
   - ○ ungulates
   - ○ pangolins
   - ○ poultry/other fowl
   - ○ goats/sheep
   - ○ camels
   - ○ swine
   - ○ cattle/buffalo
   - ○ dogs
   - ○ cats
   - ○ none (skip to question 7)

5. How are live animals stored at night? Select all that apply.
   - ○ multiple species in one enclosure
   - ○ individual species in one enclosure
   - ○ both multiple and individual species in enclosures

6. Is there a quarantine period for new animals?
   - ○ yes
   - ○ no

7. Is there on-site food production?
   - ○ yes
   - ○ no

8. If yes, who pays for the cost to grow the food crops?
   - ○ the company
   - ○ the workers

9. Is there meat available for consumption?
   - ○ yes
   - ○ no

example only, do not use in field

243848-502267

Participant ID

(For reference only)

10. If yes, where does the meat come from?
Select all that apply.
- farmed onsite
- purchased from wholesale market
- farmed and purchased from nearby local communities
- locally caught/hunted
- bought frozen
- don't know

11. Is it possible to consume bushmeat/wild animal meat on or near the site?
- yes
- no

12. Do you have special protective equipment (Example: shoes, masks, gloves) only worn at work?
- yes
- no (skip to question 15)

13. If yes, which protective equipment?
Select all that apply.
- shoes/boots
- mask
- clothes
- gloves
- gown/apron

14. When do you use protective equipment?
Select all that apply.
- handling animals
- slaughter
- butcher
- always on at work
- other: ____________

15. Do you always use disinfectant to clean?
- yes
- no

16. If yes, do you always use disinfectants to clean the following:
Select all that apply.
- animal enclosures
- food bins
- counter tops
- slaughtering/butchering equipment
- hands
- special protective equipment
- floors

17. How often are the animal enclosures cleaned?
Select one option.
Skip this question if answered "none" to question 4.
- daily
- weekly
- monthly
- as needed
- never

18. When slaughtering/butchering animals, what happens to the viscara (blood, organs, skin, sinews, etc)?
Select all that apply.
- sell
- throw into refuse bin
- throw into the street/gutter
- take home to eat
- feed to animals
- no onsite slaughter

19. Is there a designated area for the disposal of animal waste?
- yes
- no

20. If yes, do people use the dedicated area for animal waste?
- yes
- no

example only do not use in field

243849-502267

Participant ID ______________
(For reference only)

21. Since this time last year, have the animals received veterinary care? ◯ yes ◯ no
Skip to question 22 if answered "none" to question 4.

22. Since this time last year, has an animal health official inspected your animals? ◯ yes ◯ no

If answered "none" to question 4, the questionnaire is complete.

23. What do you do when an animal gets sick? Select all that apply.
- ◯ kill the animal and dispose of the carcass
- ◯ kill the animal and sell it
- ◯ sell the live animal for discounted price
- ◯ nothing different
- ◯ get veterinary care
- ◯ report to authorities
- ◯ other: ______________________

24. Since this time last year, has anyone quarantined or destroyed your animals because of infection or disease? ◯ yes ◯ no

25. If yes, which animals?
Select all that apply.
- ◯ rodents/shrews
- ◯ bats
- ◯ non-human primates
- ◯ birds
- ◯ carnivores
- ◯ ungulates
- ◯ pangolins
- ◯ poultry/other fowl
- ◯ goats/sheep
- ◯ camels
- ◯ swine
- ◯ cattle/buffalo
- ◯ dogs
- ◯ cats

26. Since this time last year, has there been a disease outbreak among any raised animals or livestock? ◯ yes ◯ no

27. If yes, which animals? (Indicate the percentage that died during the outbreak.)
Select all that apply.

	1-25%	26-50%	51-75%	76-100%	don't know
rodents/shrews	◯	◯	◯	◯	◯
bats	◯	◯	◯	◯	◯
non-human primates	◯	◯	◯	◯	◯
birds	◯	◯	◯	◯	◯
carnivores	◯	◯	◯	◯	◯
ungulates	◯	◯	◯	◯	◯
pangolins	◯	◯	◯	◯	◯
poultry/other fowl	◯	◯	◯	◯	◯
goats/sheep	◯	◯	◯	◯	◯
camels	◯	◯	◯	◯	◯
swine	◯	◯	◯	◯	◯
cattle/buffalo	◯	◯	◯	◯	◯
dogs	◯	◯	◯	◯	◯
cats	◯	◯	◯	◯	◯

example only do not use in field

243850-502267

Participant ID

(For reference only)

28. Do any animals raid or destroy food supplies?

- yes
- no

29. If yes, what animals?
Select all that apply.

- rodents/shrews
- bats
- non-human primates
- birds
- carnivores
- ungulates
- pangolins
- poultry/other fowl
- goats/sheep
- camels
- swine
- cattle/buffalo
- dogs
- cats

30. What is done to stop animals from raiding or destroying food supplies?
Select all that apply.

- barriers around fields
- barriers on individual trees
- fire
- poison
- traps
- shooting
- loud sounds
- domestic/guardian animals
- flooding
- chasing animals out
- nothing

Notes:

example only do not use in field

243851-502267
