# Supplementary material for: Socializing One Health: an innovative strategy to investigate social and behavioral risks of emerging viral threats
Source: One Health Outlook. 2021 May 14;3:11. doi: 10.1186/s42522-021-00036-9 (PMC8122533; doi:10.1186/s42522-021-00036-9)
Supplement: Supplementary file 1 — Additional file 1. Human questionnaire administered by 24 countries as part of the human surveillance scope. [file 42522_2021_36_MOESM1_ESM.zip › Socializing One Health Surveys/NaturalAreasR1.pdf]

Natural Areas Module

EIDITH

V 1.2

Add Site and Event Form ID:

0	1	2	3	4	5	6	7	8	9
0	1	2	3	4	5	6	7	8	9
0	1	2	3	4	5	6	7	8	9
0	1	2	3	4	5	6	7	8	9
0	1	2	3	4	5	6	7	8	9
0	1	2	3	4	5	6	7	8	9

Site name and date:

____

(For reference only)

1. Is this a protected area?
   - ☐ yes
   - ☐ no

2. If yes above, what is the approximate size of protected area in square kilometers? ______________________

3. If yes above, what is the name of the protected area? ______________________

4. What is the type of setting? Select all that apply.
   - ☐ forest
   - ☐ grassland
   - ☐ riparian/river/stream/lake
   - ☐ wetland
   - ☐ estuary
   - ☐ cave
   - ☐ desert/arid
   - ☐ urban park/garden

5. What are the human impacts in the natural area? Select all that apply.
   - ☐ hunting
   - ☐ tourism/recreational
   - ☐ land clearing for logging
   - ☐ land clearing for infrastructure development (including road building, dams)
   - ☐ land clearing for other extractive industry (eg mining, oil and gas, precious metals)
   - ☐ land clearing for domesticated animal production
   - ☐ land clearing for crop production
   - ☐ temporary human settlement
   - ☐ permanent human settlement
   - ☐ displaced people due to nearby conflict
   - ☐ small scale harvest of food, firewood, medicine, etc for personal/home use
   - ☐ no apparent human impact

example only do not use in field

110903-48599
