# Supplementary material for: Socializing One Health: an innovative strategy to investigate social and behavioral risks of emerging viral threats
Source: One Health Outlook. 2021 May 14;3:11. doi: 10.1186/s42522-021-00036-9 (PMC8122533; doi:10.1186/s42522-021-00036-9)
Supplement: Supplementary file 1 — Additional file 1. Human questionnaire administered by 24 countries as part of the human surveillance scope. [file 42522_2021_36_MOESM1_ESM.zip › Socializing One Health Surveys/HumanWildlifeRestaurantR1.pdf]

EIDITH V 1.0

0 1 2 3 4 5 6 7 8 9
0 1 2 3 4 5 6 7 8 9
0 1 2 3 4 5 6 7 8 9
0 1 2 3 4 5 6 7 8 9
0 1 2 3 4 5 6 7 8 9
0 1 2 3 4 5 6 7 8 9

Add Human Questionnaire Form ID

Participant ID ____________________
(For reference only)

1. Do you live on site?
   - ☐ yes
   - ☐ no

2. To the best of your knowledge, how many people work at this site?
   - ☐ <10
   - ☐ 11-50
   - ☐ 51-100
   - ☐ 101-1000
   - ☐ >1001

3. How long have you worked here?
   Select one option.
   - ☐ <1 month
   - ☐ 1 month - 1 year
   - ☐ >1 year - 5 years
   - ☐ >5 years

4. What wild animals are on the menu today?
   Select all that apply.
   - ☐ rodents/shrews
   - ☐ bats
   - ☐ non-human primates
   - ☐ birds
   - ☐ carnivores
   - ☐ ungulates
   - ☐ pangolins

5. Since this time last year, have you had live animals on site?
   - ☐ yes
   - ☐ no

6. If yes, where do the animals come from?
   Select all that apply.
   - ☐ farmed and/or purchased from nearby local communities
   - ☐ wholesale live animal market
   - ☐ locally caught/hunted
   - ☐ other: ____________________

7. How do the live animals get to the restaurant?
   Select all that apply.
   - ☐ transport truck
   - ☐ car
   - ☐ motorbike
   - ☐ cart
   - ☐ delivered by hunter
   - ☐ public bus
   - ☐ brought in by customer
   - ☐ other: ____________________

example only do not use in field

107397-961687

8. How are live animals stored at night?
   Select all that apply.
   - multiple species in one enclosure
   - individual species in one enclosure
   - both multiple and individual species in enclosures

9. Are live animals slaughtered at the restaurant?
   - yes
   - no

10. Do you have special protective equipment (Example: shoes, masks, gloves) only worn at work?
    - yes
    - no

11. If yes, which protective equipment?
    Select all that apply.
    - shoes/boots
    - mask
    - clothes
    - gloves
    - gown/apron

12. When do you use protective equipment?
    Select all that apply.
    - handling animals
    - slaughter
    - butcher
    - always on at work
    - other: ______________________

13. Do you always use disinfectant to clean?
    - yes
    - no

14. If yes, do you always use disinfectants to clean the following:
    Select all that apply.
    - animal enclosures
    - food bins
    - counter tops
    - slaughtering/butchering equipment
    - hands
    - special protective equipment
    - floors

15. Is there a designated area for rubbish, including animal waste from slaughter/butcher and animal excrement?
    - yes
    - no

16. If yes, do people use the dedicated location for rubbish?
    - yes
    - no

example only do not use in field

107398-961687

17. Do any animals raid food or destroy supplies?
- yes
- no

18. If yes, which animals? Select all that apply.
- rodents/shrews
- bats
- non-human primates
- birds
- carnivores
- ungulates
- pangolins
- poultry/other fowl
- goats/sheep
- camels
- swine
- cattle/buffalo
- dogs
- cats

19. What is done to stop animals from raiding or destroying food supplies? Select all that apply.
- barriers around fields
- barriers on individual trees
- fire
- poison
- traps
- shooting
- loud sounds
- domestic/guardian animals
- flooding
- chasing animals out
- nothing

example only do not use in field
