# Supplementary material for: Socializing One Health: an innovative strategy to investigate social and behavioral risks of emerging viral threats
Source: One Health Outlook. 2021 May 14;3:11. doi: 10.1186/s42522-021-00036-9 (PMC8122533; doi:10.1186/s42522-021-00036-9)
Supplement: Supplementary file 1 — Additional file 1. Human questionnaire administered by 24 countries as part of the human surveillance scope. [file 42522_2021_36_MOESM1_ESM.zip › Socializing One Health Surveys/HumanIllnessR1.pdf]

Human Illness Module

EIDITH
V 1.4

Add Human Questionnaire Form ID

0	1	2	3	4	5	6	7	8	9
0	1	2	3	4	5	6	7	8	9
0	1	2	3	4	5	6	7	8	9
0	1	2	3	4	5	6	7	8	9
0	1	2	3	4	5	6	7	8	9
0	1	2	3	4	5	6	7	8	9

Participant ID: ____________________

A. Patient Medical Information

1. Clinical diagnosis as determined by physician or health care provider: (from medical record if available)

____

____

2. Date of hospitalization for present illness (if applicable): ____________________

3. Provide the specimen ID and the date of the specimen collection for PREDICT protocols:

Specimen ID	Date Collected
____________________	____________________
____________________	____________________
____________________	____________________
____________________	____________________
____________________	____________________
____________________	____________________

4. Antiviral use for present illness at the time of specimen collection?
   - ◯ yes
   - ◯ no

5. If yes, which medication? ____

6. Body temperature at the time of sampling (celsius) __________

B. General Patient Information (ask patient, but compare answers with medical record, if available)

7. For how many days have you had a fever? __________

8. Date of symptom onset: ____________________

9. Signs and symptoms of present illness.
   Select all that apply.

- ◯ abdominal pain
- ◯ altered consciousness
- ◯ any movement in stomach
- ◯ asphyxiate
- ◯ bleeding
- ◯ chills
- ◯ cold
- ◯ convulsions
- ◯ cough
- ◯ dark urine
- ◯ diarrhea
- ◯ dizziness
- ◯ eye pain
- ◯ fever
- ◯ headache
- ◯ jaundice
- ◯ joint pain
- ◯ malaise
- ◯ muscle pain
- ◯ no appetite
- ◯ rash
- ◯ sore throat
- ◯ stiff neck
- ◯ vomiting
- ◯ other: ______________________________
- ◯ Do not use. For future use.

example only do not use in field

249407-267368

Participant ID: ____________________
(for reference only)

10. Presence of chronic pre-existing medical illnesses:
Select all that apply.

- ○ chronic respiratory disease
- ○ asthma
- ○ diabetes
- ○ chronic cardiac disease
- ○ chronic neurological disease
- ○ chronic neuromuscular disease
- ○ hematological disorders
- ○ immunodeficiency disorders
- ○ HIV or AIDS
- ○ none
- ○ other: ____________________
- ○ Do not use. For future use.

Women Only (Questions 11 & 12)

11. Pregnancy status
- ○ yes
- ○ no
- ○ don't know

12. If yes, how many months pregnant? __________

13. Other symptom in family member or member of household?
- ○ yes
- ○ no

14. If yes, whom and what symptom?
(use symptom list from question 9)

____

____

____

____

____

C. Additional Testing (if available)

15. Lab Tests and Results
(For rapid diagnostic test include which test used)

		Specimen Collection Date	Test Result
Influenza virus	○ RT-PCR		
	○ virus culture		
	○ serology		
	○ other: ________		
Dengue virus	○ RT-PCR		
	○ virus culture		
	○ serology		
	○ other: ________		
Chikungunya virus	○ RT-PCR		
	○ virus culture		
	○ serology		
	○ other: ________		
Malaria	○ serology		
	○ microscopy		
	○ rapid diagnostic test: ________		
	○ other: ________		

example only do not use in field

249408-267368

Participant ID: ____________
(for reference only)

15. Lab Tests and Results - Continued

(For rapid diagnostic test include which test used.)		Specimen Collection Date	Test Result
other: ________	◯ RT-PCR	________	________
	◯ virus culture	________	________
	◯ serology	________	________
	◯ rapid diagnostic test: ________	________	________
	◯ other: ________	________	________
other: ________	◯ RT-PCR	________	________
	◯ virus culture	________	________
	◯ serology	________	________
	◯ rapid diagnostic test: ________	________	________
	◯ other: ________	________	________
other: ________	◯ RT-PCR	________	________
	◯ virus culture	________	________
	◯ serology	________	________
	◯ rapid diagnostic test: ________	________	________
	◯ other: ________	________	________

example only do not use in field
