# Supplementary material for: Socializing One Health: an innovative strategy to investigate social and behavioral risks of emerging viral threats
Source: One Health Outlook. 2021 May 14;3:11. doi: 10.1186/s42522-021-00036-9 (PMC8122533; doi:10.1186/s42522-021-00036-9)
Supplement: Supplementary file 1 — Additional file 1. Human questionnaire administered by 24 countries as part of the human surveillance scope. [file 42522_2021_36_MOESM1_ESM.zip › Socializing One Health Surveys/HumanHunterR1.pdf]

Hunter Module

EIDITH
v1.2

0	1	2	3	4	5	6	7	8	9
0	1	2	3	4	5	6	7	8	9
0	1	2	3	4	5	6	7	8	9
0	1	2	3	4	5	6	7	8	9
0	1	2	3	4	5	6	7	8	9
0	1	2	3	4	5	6	7	8	9

Add Human Questionnaire Form ID

Participant ID ____________________

(For reference only)

1. What animals have you hunted since this time last year?
   Select all that apply.

- ○ rodents/shrews
- ○ bats
- ○ non-human primates
- ○ birds
- ○ carnivores
- ○ ungulates
- ○ pangolins
- ○ none

If "none" selected, the questionnaire is complete.

2. Since this time last year, what methods have you used to hunt/trap animals?
   Select all that apply.

- ○ snare
- ○ bow
- ○ hands
- ○ gun
- ○ machete
- ○ knife
- ○ net
- ○ cage
- ○ trap
- ○ other: ____________________

3. What is the purpose of your trapping or hunting?
   Select all that apply.

	for consumption at home	for use of animal products at home	for sale for consump -tion	for sale alive at market	for sale of animal products	live trapping of nuisance animals for translocation	culling of nuisance animals
rodents/shrews	○	○	○	○	○	○	○
bats	○	○	○	○	○	○	○
non-human primates	○	○	○	○	○	○	○
birds	○	○	○	○	○	○	○
carnivores	○	○	○	○	○	○	○
ungulates	○	○	○	○	○	○	○
pangolins	○	○	○	○	○	○	○

example only do not use in field

Since this time last year, when you hunt or trap:

4. Are you exposed to blood?
   - ○ yes
   - ○ no

5. Have you been scratched or bitten?
   - ○ yes
   - ○ no

6. Since this time last year, have you seen an outbreak of dead wild animals?
   - ○ yes
   - ○ no

7. If yes, which wild animals?
   Select all that apply.

- ○ rodents/shrews
- ○ bats
- ○ non-human primates
- ○ birds
- ○ carnivores
- ○ ungulates
- ○ pangolins

294920-857798

Participant ID

____

(For reference only)

8. What do you do when you find an animal dead (not in a trap or shot by another hunter)?
   Select all that apply.
   - ○ touch it to see if it is still fresh
   - ○ butcher in the forest
   - ○ smoke or cook in the forest
   - ○ take home to prepare
   - ○ bury it
   - ○ report it to authorities
   - ○ take it to sell it
   - ○ nothing
   - ○ other: ______________

9. How do you transport a dead animal, if you take it?
   Select all that apply.
   - ○ not wrapped
   - ○ wrapped in leaves or other natural material
   - ○ wrapped in plastic
   - ○ in a bag
   - ○ in a basket

10. Do you have special protective equipment (Example: shoes, masks, gloves)?
    - ○ yes
    - ○ no

11. If yes, which protective equipment?
    Select all that apply.
    - ○ shoes/boots
    - ○ mask
    - ○ clothes
    - ○ gloves
    - ○ gown/apron

12. When do you use protective equipment?
    Select all that apply.
    - ○ handling animals
    - ○ slaughter
    - ○ butcher
    - ○ always on at work
    - ○ other: ______________

example only do not use in field

294921-857798
