# Supplementary material for: Socializing One Health: an innovative strategy to investigate social and behavioral risks of emerging viral threats
Source: One Health Outlook. 2021 May 14;3:11. doi: 10.1186/s42522-021-00036-9 (PMC8122533; doi:10.1186/s42522-021-00036-9)
Supplement: Supplementary file 1 — Additional file 1. Human questionnaire administered by 24 countries as part of the human surveillance scope. [file 42522_2021_36_MOESM1_ESM.zip › Socializing One Health Surveys/HumanTempSettlementR1.pdf]

Temporary Settlement Module

EIDITH
v1.1

0	1	2	3	4	5	6	7	8	9
0	1	2	3	4	5	6	7	8	9
0	1	2	3	4	5	6	7	8	9
0	1	2	3	4	5	6	7	8	9
0	1	2	3	4	5	6	7	8	9
0	1	2	3	4	5	6	7	8	9

Add Human Questionnaire Form ID

Participant ID ____________________

(For reference only)

1. What is your nationality? ____________________

2. How long have you lived at this settlement?
   Select one option.
   - ○ <1 week
   - ○ 1-4 weeks
   - ○ 1-5 months
   - ○ 6-11 months
   - ○ > 1 year
   - ○ entire life

3. To the best of your knowledge, how many people live at this site?
   Select one option.
   - ○ <10
   - ○ 10-100
   - ○ 101-1000
   - ○ 1001-10,000
   - ○ >10,000

4. Are all the people living here from this country?
   - ○ yes
   - ○ no

5. Why did you settle here?
   Select all that apply.
   - ○ job/work (voluntary relocation)
   - ○ family (voluntary relocation)
   - ○ marriage (voluntary relocation)
   - ○ conflict (forced relocation)
   - ○ dispossession of previous home (forced relocation)
   - ○ natural disaster (forced relocation)

6. Is there on-site food production?
   - ○ yes
   - ○ no

7. Is there meat available for consumption?
   - ○ yes
   - ○ no

8. If yes, where does the meat come from?
   Select all that apply.
   - ○ farmed onsite
   - ○ farmed and purchased from nearby local communities
   - ○ purchased from wholesale market
   - ○ locally caught/hunted
   - ○ bought frozen
   - ○ don't know

9. Question removed

example only do not use in field

294498-400443

Participant ID

____

(For reference only)

10. Which animals are available for consumption?
 This question is required if answered "yes" to Q7.

 Select all that apply.
 - ◯ rodents/shrews
 - ◯ bats
 - ◯ non-human primates
 - ◯ birds
 - ◯ carnivores
 - ◯ ungulates
 - ◯ pangolins
 - ◯ poultry/other fowl
 - ◯ goats/sheep
 - ◯ camels
 - ◯ swine
 - ◯ cattle/buffalo
 - ◯ dogs
 - ◯ cats

11. Is there a designated area for rubbish, including animal waste from slaughter/butcher and animal excrement?
 - ◯ yes
 - ◯ no

12. If yes, do people use the designated location for rubbish?
 - ◯ yes
 - ◯ no

13. Do any animals raid food supplies or destroy crops?
 - ◯ yes
 - ◯ no

14. If yes, which animals?
 Select all that apply.
 - ◯ rodents/shrews
 - ◯ bats
 - ◯ non-human primates
 - ◯ birds
 - ◯ carnivores
 - ◯ ungulates
 - ◯ pangolins
 - ◯ poultry/other fowl
 - ◯ goats/sheep
 - ◯ camels
 - ◯ swine
 - ◯ cattle/buffalo
 - ◯ dogs
 - ◯ cats

15. What is done to stop animals from raiding or destroying food supplies?
 Select all that apply.
 - ◯ barriers around fields
 - ◯ barriers on individual trees
 - ◯ fire
 - ◯ poison
 - ◯ traps
 - ◯ shooting
 - ◯ loud sounds
 - ◯ domestic/guardian animals
 - ◯ flooding
 - ◯ chasing animals out
 - ◯ nothing

example only do not use in field

294499-400443
