# Supplementary material for: Socializing One Health: an innovative strategy to investigate social and behavioral risks of emerging viral threats
Source: One Health Outlook. 2021 May 14;3:11. doi: 10.1186/s42522-021-00036-9 (PMC8122533; doi:10.1186/s42522-021-00036-9)
Supplement: Supplementary file 1 — Additional file 1. Human questionnaire administered by 24 countries as part of the human surveillance scope. [file 42522_2021_36_MOESM1_ESM.zip › Socializing One Health Surveys/HumanMarketValueChainR1.pdf]

EIDITH
V 1.0

0 1 2 3 4 5 6 7 8 9
0 1 2 3 4 5 6 7 8 9
0 1 2 3 4 5 6 7 8 9
0 1 2 3 4 5 6 7 8 9
0 1 2 3 4 5 6 7 8 9
0 1 2 3 4 5 6 7 8 9

Add Human Questionnaire Form ID

Participant ID ____________________
(For reference only)

1. Do you live on the work site?
   - yes
   - no

2. To the best of your knowledge, how many people work at this site?
   Select one option.
   - <10
   - 10-50
   - 51-100
   - 101-1000
   - >1001

3. How long have you worked at this market?
   Select one option.
   - <1 month
   - 1 month - 1 year
   - >1 year - 5 years
   - >5 years

4. What animals are you selling today?
   Select all that apply.
   - rodents/shrews
   - bats
   - non-human primates
   - birds
   - carnivores
   - ungulates
   - pangolins
   - poultry/other fowl
   - goats/sheep
   - camels
   - swine
   - cattle/buffalo
   - dogs
   - cats

5. Have you sold a live animal today?
   - yes
   - no

6. Who buys the live animals you sell?
   Select all that apply.
   - customer for home use
   - restaurants/hotels
   - customer who resells at another market
   - other: ____________________

7. Where do the animals come from?
   Select all that apply.
   - farmed and/or purchased from nearby local communities
   - wholesale live animal market
   - locally caught/hunted
   - other: ____________________

8. How do the live animals get to the market?
   Select all that apply.
   - transport truck
   - car
   - motorbike
   - cart
   - delivered by hunter
   - public bus
   - other: ____________________

example only do not use in field

172611-560664

9. How are live animals stored at night?
   Select all that apply.
   - ○ multiple species in one enclosure
   - ○ individual species in one enclosure
   - ○ both multiple and individual species in enclosures

10. How long do you keep alive animals before you sell them? (in hours) ____________

11. What do you do when an animal gets sick?
    Select all that apply.
    - ○ kill the animal and dispose of the carcass
    - ○ kill the animal and sell it
    - ○ sell the live animal for discounted price
    - ○ nothing different
    - ○ get veterinary care
    - ○ report to authorities
    - ○ other: ____________________________

12. Do you have special protective equipment (Example: shoes, masks, gloves) only worn at work?
    - ○ yes
    - ○ no

13. If yes, which protective equipment?
    Select all that apply.
    - ○ shoes/boots
    - ○ mask
    - ○ clothes
    - ○ gloves
    - ○ gown/apron

14. When do you use protective equipment?
    Select all that apply.
    - ○ handling animals
    - ○ slaughter
    - ○ butcher
    - ○ always on at work
    - ○ other: ____________________

15. Is protective equipment used every time an animal is handled?
    - ○ yes
    - ○ no

16. When butchering animals, what happens to the refuse (blood, organs, skin, sinews, etc)?
    Select all that apply.
    - ○ sell
    - ○ throw into refuse bin
    - ○ throw into the street/gutter
    - ○ take home to eat
    - ○ feed to animals
    - ○ no onsite slaughter

17. Do you always use disinfectant to clean?
    - ○ yes
    - ○ no

18. If yes, do you always use disinfectants to clean the following:
    Select all that apply.
    - ○ animal enclosures
    - ○ food bins
    - ○ counter tops
    - ○ slaughtering/butchering equipment
    - ○ hands
    - ○ special protective equipment
    - ○ floors

example only do not use in field

19. How often are the animal enclosures cleaned?
    Select one option.
    - daily
    - weekly
    - monthly
    - as needed
    - never

20. Is there a designated area for rubbish, including animal waste from slaughter/butcher and animal excrement?
    - yes
    - no

21. If yes, do people use the dedicated area for rubbish?
    - yes
    - no

22. How often does the market close?
    Select one option.
    - once per week
    - once every 2 weeks
    - once per month
    - as needed
    - only operates 1-5 days per week
    - never

23. Since this time last year, has an animal health official inspected your animals?
    - yes
    - no

24. Since this time last year, has anyone destroyed your animals because of infection or disease?
    - yes
    - no

25. If yes, which animals?
    Select all that apply.
    - rodents/shrews
    - bats
    - non-human primates
    - birds
    - carnivores
    - ungulates
    - pangolins
    - poultry/other fowl
    - goats/sheep
    - camels
    - swine
    - cattle/buffalo
    - dogs
    - cats

26. What is done to stop animals from raiding or destroying food supplies?
    Select all that apply.
    - barriers around fields
    - barriers on individual trees
    - fire
    - poison
    - traps
    - shooting
    - loud sounds
    - domestic/guardian animals
    - flooding
    - chasing animals out
    - nothing

example only do not use in field
