# Supplementary material for: Socializing One Health: an innovative strategy to investigate social and behavioral risks of emerging viral threats
Source: One Health Outlook. 2021 May 14;3:11. doi: 10.1186/s42522-021-00036-9 (PMC8122533; doi:10.1186/s42522-021-00036-9)
Supplement: Supplementary file 1 — Additional file 1. Human questionnaire administered by 24 countries as part of the human surveillance scope. [file 42522_2021_36_MOESM1_ESM.zip › Socializing One Health Surveys/WildlifeRestaurantR1.pdf]

Wildlife Restaurant Module

EIDITH

V 1.2

Add Site and Event Form ID:

0	1	2	3	4	5	6	7	8	9
0	1	2	3	4	5	6	7	8	9
0	1	2	3	4	5	6	7	8	9
0	1	2	3	4	5	6	7	8	9
0	1	2	3	4	5	6	7	8	9
0	1	2	3	4	5	6	7	8	9

Site name and date:

____

(For reference only)

1. How would you best describe this restaurant? Select one option.
   - ◯ permanent structure
   - ◯ mobile/semi-permanent structure

2. How many chairs are there at the restaurant (inside and outside)? ________________

3. Which taxonomic groups are sold at this restaurant and for what purpose? Select all that apply for each row.

	offered for consumption	held live	sold live	products sold	slaughtered	none observed
rodents/shrews	◯	◯	◯	◯	◯	◯
bats	◯	◯	◯	◯	◯	◯
non-human primates	◯	◯	◯	◯	◯	◯
birds	◯	◯	◯	◯	◯	◯
carnivores	◯	◯	◯	◯	◯	◯
ungulates	◯	◯	◯	◯	◯	◯
pangolins	◯	◯	◯	◯	◯	◯

4. What type of animal waste is present at the site/event? Select all that apply.
   - ◯ feces
   - ◯ soiled bedding (urine and feces)
   - ◯ animal tissue and/or blood
   - ◯ none

5. Is there a designated area for slaughtering and/or butchering animals? Select one option.
   - ◯ yes, separate from dining area
   - ◯ yes, same as dining area
   - ◯ no

6. What types of biosecurity measures are practiced at the facility? Select all that apply.
   - ◯ hand washing facilities
   - ◯ gloves for personnel handling animals or raw animal products
   - ◯ protective clothing and footwear for personnel performing butchering and slaughtering
   - ◯ washing and disinfecting animal crates and equipment in contact with animals or animal products
   - ◯ removal of sick or dead animals from live animal settings
   - ◯ no biosecurity observed

example only do not use in field

112000-988333
