# Supplementary material for: Socializing One Health: an innovative strategy to investigate social and behavioral risks of emerging viral threats
Source: One Health Outlook. 2021 May 14;3:11. doi: 10.1186/s42522-021-00036-9 (PMC8122533; doi:10.1186/s42522-021-00036-9)
Supplement: Supplementary file 1 — Additional file 1. Human questionnaire administered by 24 countries as part of the human surveillance scope. [file 42522_2021_36_MOESM1_ESM.zip › Socializing One Health Surveys/HumanSpecimenR1.pdf]

Human Specimen Data

EIDITH V 1.0

Add Human Questionnaire Form ID:

0	1	2	3	4	5	6	7	8	9
0	1	2	3	4	5	6	7	8	9
0	1	2	3	4	5	6	7	8	9
0	1	2	3	4	5	6	7	8	9
0	1	2	3	4	5	6	7	8	9
0	1	2	3	4	5	6	7	8	9

1. Date of Specimen Collection ________________

2. Time of Specimen Collection ________________

3. Specimen was originally collected for diagnostic purposes.
   - yes
   - no

4. Specimen ID: ______________________________

5. Participant ID: ______________________________

6. Specimen Type - Select one option.

bile	blood clot	oral swab	rectal swab
cerebrospinal fluid	blood (whole)	nasal swab	feces
milk	buffy coat	nasopharyngeal swab	preputial swab
pericardial fluid	plasma	ocular swab	urine/urogenital swab
peritoneal fluid	red blood cells	oropharyngeal swab	vaginal swab
pleural fluid	serum	saliva	other: ________________
urine	for future use - do not use	sputum	for future use - do not use

7. Specimen Medium
   - trizol
   - viral transport media (VTM)
   - 10% buffered formalin
   - RNA later
   - lysis buffer
   - no medium
   - other: ________________

8. Specimen Container
   - cryotube
   - glass jar
   - plastic container
   - whirl pack
   - other: ________________

9. Specimen Storage Method
   - ultra low freezer (-80c)
   - freezer (-20c to -40c)
   - liquid nitrogen
   - room temperature
   - other: ________________

10. Storage facility ______________________________

11. Storage location within facility ______________________________
(final destination)

234583-842155

example only do not use in field
