# Supplementary material for: Socializing One Health: an innovative strategy to investigate social and behavioral risks of emerging viral threats
Source: One Health Outlook. 2021 May 14;3:11. doi: 10.1186/s42522-021-00036-9 (PMC8122533; doi:10.1186/s42522-021-00036-9)
Supplement: Supplementary file 1 — Additional file 1. Human questionnaire administered by 24 countries as part of the human surveillance scope. [file 42522_2021_36_MOESM1_ESM.zip › Socializing One Health Surveys/HumanTourismR1.pdf]

Tourism Module

EIDITH
V 1.0

0	1	2	3	4	5	6	7	8	9
0	1	2	3	4	5	6	7	8	9
0	1	2	3	4	5	6	7	8	9
0	1	2	3	4	5	6	7	8	9
0	1	2	3	4	5	6	7	8	9
0	1	2	3	4	5	6	7	8	9

Add Human Questionnaire Form ID

Participant ID ____________________
(For reference only)

1. What is your nationality? ____________________

2. List all the countries you have visited in the past month.

____________________ ____________________

____________________ ____________________

____________________ ____________________

3. How long have you been at the current location?
   Select one option.
   - ◯ 1 day
   - ◯ 2-7 days
   - ◯ 8-14 days
   - ◯ > 2 weeks

4. Which activities did you undertake on this trip?
   Select all that apply.
   - ◯ wildlife tourism
   - ◯ cave tourism
   - ◯ camping
   - ◯ hunting
   - ◯ trekking in natural areas
   - ◯ religious pilgrimage
   - ◯ visiting wildlife market
   - ◯ other: ____________________

5. What animals have you eaten during this trip?
   Select all that apply.
   - ◯ rodents/shrews
   - ◯ bats
   - ◯ non-human primates
   - ◯ birds
   - ◯ carnivores
   - ◯ ungulates
   - ◯ pangolins
   - ◯ poultry/other fowl
   - ◯ goats/sheep
   - ◯ camels
   - ◯ swine
   - ◯ cattle/buffalo
   - ◯ dogs
   - ◯ cats
   - ◯ unknown
   - ◯ none

106775-93972
