# Supplementary material for: Socializing One Health: an innovative strategy to investigate social and behavioral risks of emerging viral threats
Source: One Health Outlook. 2021 May 14;3:11. doi: 10.1186/s42522-021-00036-9 (PMC8122533; doi:10.1186/s42522-021-00036-9)
Supplement: Supplementary file 1 — Additional file 1. Human questionnaire administered by 24 countries as part of the human surveillance scope. [file 42522_2021_36_MOESM1_ESM.zip › Socializing One Health Surveys/ExtractIndustryR1.pdf]

Extractive Industry Site Module

EIDITH

V 1.5

Add Site and Event Form ID: ⓪ ① ② ③ ④ ⑤ ⑥ ⑦ ⑧ ⑨ (six rows)

Site name and date: ____________________

(For reference only)

1. Is this an existing or planned extractive industry site? Select one option per row.

	none	existing	planned
coal	○	○	○
coltan	○	○	○
diamond or other gemstone	○	○	○
electricity	○	○	○
tin	○	○	○
gold/silver	○	○	○
lead	○	○	○
oil/gas	○	○	○
timber/plant	○	○	○
other: ____________	○	○	○

2. For each extractive industry at the site, what was the state of land before conversion? Select all that apply.

	undisturbed habitat	protected area	savannah or grassland	agricultural fields	unknown	other: ____________	
coal	○	○	○	○	○	○	
coltan	○	○	○	○	○	○	
diamond or other gemstone	○	○	○	○	○	○	
electricity	○	○	○	○	○	○	
tin	○	○	○	○	○	○	
gold/silver	○	○	○	○	○	○	
lead	○	○	○	○	○	○	
oil/gas	○	○	○	○	○	○	
timber/plant	○	○	○	○	○	○	
other: ____________	○	○	○	○	○	○	

3. Which of the following are used to move the resource (e.g., timber, ore, oil) and/or staff to and from the facility? Select all that apply.

- ○ road
- ○ rail
- ○ airstrip
- ○ port
- ○ river
- ○ helipad
- ○ none

4. Are roads being built at the site?

- ○ yes
- ○ no

5. Is there a place where meals are provided or food is sold?

- ○ yes
- ○ no

6. Is there an on-site healthcare facility available to workers?

- ○ yes
- ○ no

7. If yes, do they have:

	yes	no
capacity for medical treatment?	○	○
capacity for preventive services?	○	○
a posted ebola or other disease outbreak preparedness plan?	○	○
capacity for pharmacy services?	○	○

example only do not use in field

110583-442655
