# Supplementary material for: Socializing One Health: an innovative strategy to investigate social and behavioral risks of emerging viral threats
Source: One Health Outlook. 2021 May 14;3:11. doi: 10.1186/s42522-021-00036-9 (PMC8122533; doi:10.1186/s42522-021-00036-9)
Supplement: Supplementary file 1 — Additional file 1. Human questionnaire administered by 24 countries as part of the human surveillance scope. [file 42522_2021_36_MOESM1_ESM.zip › Socializing One Health Surveys/HumanCropProductionR1.pdf]

Crop Production Module

EIDITH
V 1.0

0 1 2 3 4 5 6 7 8 9
0 1 2 3 4 5 6 7 8 9
0 1 2 3 4 5 6 7 8 9
0 1 2 3 4 5 6 7 8 9
0 1 2 3 4 5 6 7 8 9
0 1 2 3 4 5 6 7 8 9

Add Human Questionnaire Form ID

Participant ID ______________________
(For reference only)

1. Do you live on the work site?
   - yes
   - no

2. To the best of your knowledge, how many people work at this site?
   Select one option.
   - <10
   - 10-100
   - 101-1000
   - 1001-10,000
   - >10,000

3. How long have you worked at this site?
   Select one option.
   - <1 month
   - 1 month - 1 year
   - >1 year - 5 years
   - >5 years

4. Which crops are at this site?
   Select all that apply.
   - coffee/tea/cocoa plants
   - fruit/nut trees
   - oil tree plantation
   - oil seed crops
   - dry grains
   - sugar
   - vegetable/fruit crops
   - pulses/legume
   - fiber
   - forages
   - cover crops
   - fallow fields
   - rubber

5. Does the farm use manure, guano or night soil to fertilizer the crops?
   - yes
   - no

6. If yes, which types?
   Select all that apply.
   - poultry/other fowl
   - camel
   - swine
   - cattle/buffalo
   - bird guano
   - bat guano
   - night soil

7. Is there meat available for the consumption?
   - yes
   - no

8. If yes, where does the meat come from?
   Select all that apply.
   - farmed onsite
   - farmed and purchased from nearby local communities
   - purchased from wholesale market
   - locally caught/hunted
   - bought frozen
   - don't know

9. Is it possible to consume bushmeat/wild animal meat on or near the site?
   - yes
   - no

example only do not use in field

169748-861177

10. Is there a designated area for rubbish, including animal waste from slaughter/butcher and animal excrement?
- yes
- no

11. If yes, do people use the designated location for rubbish?
- yes
- no

12. Do any animals raid food supplies or destroy crops?
- yes
- no

13. If yes, which animals?
Select all that apply.
- rodents/shrews
- bats
- non-human primates
- birds
- carnivores
- ungulates
- pangolins
- poultry/other fowl
- goats/sheep
- camels
- swine
- cattle/buffalo
- dogs
- cats

14. What is done to stop animals from raiding or destroying food supplies?
Select all that apply.
- barriers around fields
- barriers on individual trees
- fire
- poison
- traps
- shooting
- loud sounds
- domestic/guardian animals
- flooding
- chasing animals out
- nothing

example only do not use in field

169749-861177
