# Supplementary material for: Socializing One Health: an innovative strategy to investigate social and behavioral risks of emerging viral threats
Source: One Health Outlook. 2021 May 14;3:11. doi: 10.1186/s42522-021-00036-9 (PMC8122533; doi:10.1186/s42522-021-00036-9)
Supplement: Supplementary file 2 — Additional file 2. Ebola Host Project questionnaire administered in Guinea, Liberia, and Sierra Leone. [file 42522_2021_36_MOESM2_ESM.pdf]

Ebola Host Project PREDICT
Human Questionnaire

EIDITH
V 1.1

Add Site and Event Form ID: ⓪ ① ② ③ ④ ⑤ ⑥ ⑦ ⑧ ⑨ (×6 rows)

Site name:

____

(For reference only)

1. Participant ID:______________________ Consent Form Administered & Signed ◯ yes ◯ no

2. Description of Interview Location - Select all that apply.
(To be completed by interviewer prior to administrative questionnaire.
Prepare and download modules in advance.)
   - ◯ Animal Production or Abattoir Site
   - ◯ Crop Production Site
   - ◯ Extractive Industry Site
   - ◯ Market or Value Chain Site
   - ◯ Temporary Settlement Site
   - ◯ Natural Areas (eg. forest, urban park/garden)
   - ◯ Wildlife Restaurant
   - ◯ Zoos or Sanctuaries
   - ◯ Outbreak Investigation Site
   - ◯ Control Site
   - ◯ Other:______________________

3. Date of interview ______________________

4. Begin time of interview ______________
(Example: 17:50)

5. End time of interview ______________
(Example: 19:20)

6. Where are you conducting this interview?

Village/Town/City ______________ Province/State ______________

Latitude ______________________ Longitude ______________________

Interviewer: Please collect GPS coordinates if administering using paper and pen.

7. Interviewer Observed Gender
   - ◯ male
   - ◯ female
   - ◯ other

INTERVIEW/QUESTIONNAIRE BEGINS

Demographics Section (include observation question 7)

8. How old are you? ___________
If the exact age is unknown, enter the respondent's estimated age.

9. Where do you live?

Village/Town/City ______________ Province/State ______________

Latitude ______________________ Longitude ______________________

Interviewer: Probe for landmarks or nearest known site if area unknown.
GPS coordinates to be identified and entered after completion of interview.

590393-402351

10. How long have you lived there?
    Select one option.
    - ◯ <1 month
    - ◯ 1 month - 1 year
    - ◯ >1 - 5 years
    - ◯ >5 - 10 years
    - ◯ >10 years

11. How many other people live in the dwelling where you live? ________
    Skip to question 14 if answer is 0.

12. How many in the dwelling are children less than 5 years old? ________

13. How many in the dwelling are male? ________

14. How many rooms are there in the dwelling where you live? ________
    (Do not include bathroom or kitchen)

15. Is the dwelling a permanent structure (that cannot be moved)?
    - ◯ yes
    - ◯ no

Participant ID

____

(For reference only)

Livelihood Section

In this section, I'd like to ask you about education and the kinds of work activities that you have done since this time last year.

16. What is the highest level of education you have completed?
Select one option.
- ◯ primary school
- ◯ secondary school
- ◯ college/university/professional
- ◯ none

17. What is the highest level of education that your mother completed?
Select one option.
- ◯ primary school
- ◯ secondary school
- ◯ college/university/professional
- ◯ none

18. Since this time last year what are the activities you have done to earn your livelihood?
Select all that apply
- ◯ extraction of minerals, gas, oil timber
- ◯ crop production
- ◯ wildlife restaurant business
- ◯ wild/exotic animal trade/market business
- ◯ rancher/farmer animal production business
- ◯ meat processing, slaughterhouse, abattoir
- ◯ zoo/sanctuary animal health care
- ◯ protected area worker
- ◯ hunter/trapper/fisher
- ◯ forager/gatherer/non-timber forest product collector
- ◯ migrant laborer
- ◯ nurse, doctor, traditional healer, community health worker
- ◯ construction (road, housing)
- ◯ other: ______________________

19. If more than one activity was selected, what is the activity on which you spend the most time since this time last year? Select one option.
- ◯ extraction of minerals, gas, oil timber
- ◯ crop production
- ◯ wildlife restaurant business
- ◯ wild/exotic animal trade/market business
- ◯ rancher/farmer animal production business
- ◯ meat processing, slaughterhouse, abattoir
- ◯ zoo/sanctuary animal health care
- ◯ protected area worker
- ◯ hunter/trapper/fisher
- ◯ forager/gatherer/non-timber forest product collector
- ◯ migrant laborer
- ◯ nurse, doctor, traditional healer, community health worker
- ◯ construction (road, housing)
- ◯ other: ______________________

20. Which best describes your job position?
Select one option.
- ◯ manager/owner/foreman
- ◯ worker
- ◯ live and work at home independently (If chosen, skip to Medical History Section.)
- ◯ professional
- ◯ other: ________________

21. Where do you work?

Village/Town/City __________ Province/State __________

Latitude ________________ Longitude ________________

Interviewer: Probe for landmarks or nearest known site if area unknown.
GPS coordinates to be identified and entered after completion of interview.

590395-402351

Participant ID

____
(For reference only)

Medical History Section

In this section, I'm going to ask you about illness and treatment that have occurred in the community.

22. Before the Ebola outbreak, that is before 1 June 2013, where did you usually get treatment for medical problems?
Select all that apply.

- ○ clinic/health center
- ○ hospital
- ○ mobile clinic
- ○ community health worker
- ○ traditional healer
- ○ dispensary or pharmacy

23. During the Ebola outbreak, that is from 1 June 2013 through 31 March 2016, where did you usually get treatment for medical problems?
Select all that apply.

- ○ clinic/health center
- ○ hospital
- ○ mobile clinic
- ○ community health worker
- ○ traditional healer
- ○ dispensary or pharmacy

24. During the Ebola outbreak, that is from 1 June 2013 through 31 March 2016, did you have an unusual illness with any of the following symptoms?
Select all that apply. (READ ONLY SYMPTOMS)

- ○ fever with headache and severe fatigue or weakness (encephalitis)
- ○ fever with bleeding or bruising not related to injury (hemorrhagic fever)
- ○ fever with cough and shortness of breath or difficulty breathing (SARI)
- ○ fever with muscle aches, cough, or sore throat (ILI)
- ○ fever with diarrhea or vomiting
- ○ fever with rash
- ○ persistent rash or sores on skin
- ○ no (if no, skip to question 26)
- ○ yes, but none of these symptoms-describe: ______________________

25. In your opinion, when you were sick, what caused this sickness?
Select all that apply.

- ○ contact with sick people and/or their bodily fluids
- ○ contact with wild animals
- ○ contact with domestic animals and/or excretia
- ○ bad food or water
- ○ bad spirits/witchcraft
- ○ wound or injury
- ○ contact with a corpse/dead body
- ○ I don't know
- ○ other: ______________________

26. During the Ebola outbreak, that is from 1 June 2013 through 31 March 2016, were you diagnosed with Ebola?

- ○ yes
- ○ no

If No to question 24 & No to question 26, Skip to question 42
If No to question 26 & Yes to question 24, Skip to question 28

27. In your opinion, when you were sick with Ebola, what caused this sickness?
Select all that apply.

- ○ contact with sick people and/or their bodily fluids
- ○ contact with wild animals
- ○ contact with domestic animals and/or excretia
- ○ bad food or water
- ○ bad spirits/witchcraft
- ○ wound or injury
- ○ contact with a corpse/dead body
- ○ I don't know
- ○ other: ______________________

28. When did you first become sick with Ebola (or that unusual illness)? ______________________ date
I don't remember the date. ○

590396-402351

Participant ID ________________
(For reference only)

29. In total, how many days were you sick after first becoming ill? ________ days

30. In total, how many days were you sick at home? ________ days

31. While you were sick at home, did you ever isolate yourself for any period of time?
- ○ yes, from my family
- ○ yes, from both my family and my animals
- ○ yes, from my animals
- ○ no (skip to question 34)

32. How many days after becoming sick did you first isolate yourself? ________ days

33. In total, how many days did you isolated yourself? ________ days

34. What did you do after you first became sick?
Select all that apply.
- ○ I went to an Ebola Treatment Center - that is, a place specially dedicated to the care and treatment of people sick with Ebola
- ○ I went to a traditional healer
- ○ I self-medicated (i.e., took oral hydration fluid or medicine I bought)
- ○ I isolated myself in the bush/forest
- ○ other: ______________________

If no Ebola Treatment Center, skip to Question 39.

35. Why did you choose to go to an Ebola Treatment Center?
Select all that apply.
- ○ because I thought it was the right thing to do
- ○ because I was afraid I was going to die
- ○ because of proximity
- ○ because the cost of care would be covered
- ○ because of the medical resources available
- ○ because of the expertise of the Ebola Treatment Center staff
- ○ because I trusted the Ebola Treatment Center
- ○ because there was no clinical care available near where I live
- ○ because I was taken there without being asked
- ○ because the media suggested I go to the nearest health facility
- ○ other: ______________________

36. Where was that Ebola Treatment Center located or what was the name?

____
(Name city/town/zone)

37. How many days were you sick before you went to the Ebola Treatment Center? ________ days

38. When did you leave the treatment center? ____________ date

(Prompt: Do you have your discharge card? The date should be written there. If you can't remember exactly, give your best guess.)

If participant went to an Ebola Treatment Center, skip to Question 40.

39. If no, why did you chose NOT to go to an Ebola Treatment Center? Select all that apply.
- ○ because of the distance
- ○ because I thought I wasn't very sick
- ○ because I didn't think they had the resources I needed
- ○ because I didn't think they had the necessary expertise
- ○ because I did not trust the Ebola Treatment Center
- ○ I was told there was no bed available
- ○ because I was afraid of getting in the ambulance/fear that it was contaminated
- ○ because I was afraid of getting sick with Ebola at the Ebola Treatment Center
- ○ fear of being stigmatized
- ○ I was afraid my family would be quarantined
- ○ fear of dying at the Ebola Treatment Center
- ○ fear of leaving my family
- ○ other: ______________________

590397-402351

Participant ID ____________

(For reference only)

40. While you were sick with Ebola (or that unusual illness), did you have any contact with animals?
   - ◯ yes
   - ◯ no

41. If yes, which taxa? Select all that apply.
   For each taxa selected provide the number of days you had any contact with these animals while sick?

rodents/shrews	◯	☐
bats	◯	☐
non-human primates	◯	☐
birds	◯	☐
carnivores	◯	☐
ungulates	◯	☐
pangolins	◯	☐
poultry/other fowl	◯	☐
goats/sheep	◯	☐
camels	◯	☐
swine	◯	☐
cattle/buffalo	◯	☐
dogs	◯	☐
cats	◯	☐

42. During the Ebola outbreak, that is from 1 June 2013 through 31 March 2016, did anyone living at your home (i.e., children, aunts, uncles, grand-parents) become sick with Ebola?
   If no one had any symptoms, skip to the City Section.
   - ◯ yes
   - ◯ no

43. How many people living at your home became sick with Ebola? __________

Ebola Host Project PREDICT Human Questionnaire

Sick Person Matrix

Participant ID ____________________

(For reference only)

Now I am going to ask you a few questions about each person living at your home who became sick with Ebola.

Question	Response	person 1	person 2	person 3	person 4	person 5	person 6
44. Who was this person that became sick in relation to you? Select all that apply.	spouse	○	○	○	○	○	○
	child	○	○	○	○	○	○
	parent	○	○	○	○	○	○
	sibling	○	○	○	○	○	○
	grand-parent	○	○	○	○	○	○
	other: ____________	○	○	○	○	○	○
45. When did this person first become sick with Ebola? (If you can't remember exactly, give your best guess.)	date	______	______	______	______	______	______
	don't know	○	○	○	○	○	○
46. In total, how many days was this person sick after first becoming ill?	days	☐	☐	☐	☐	☐	☐
	don't know	○	○	○	○	○	○
47. In total, how many days was this person sick at home?	days	☐	☐	☐	☐	☐	☐
	don't know	○	○	○	○	○	○
48. While this person was sick at home, did he or she ever isolate him or herself for any period of time?	yes, from his or her family	○	○	○	○	○	○
	yes, from his or her animals	○	○	○	○	○	○
	yes, from both his or her family and his or her animals	○	○	○	○	○	○
(If no, skip to question 51.)	no	○	○	○	○	○	○
49. How many days after becoming sick did this person first isolate him or herself?	days	☐	☐	☐	☐	☐	☐
	don't know	○	○	○	○	○	○
50. In total, how many days did this person isolate him or herself?	days	☐	☐	☐	☐	☐	☐
	don't know	○	○	○	○	○	○
51. What did this person do after becoming sick? Select all that apply.	this person went to an Ebola Treatment Center—that is, a place specially dedicated to the care and treatment of people sick with Ebola	○	○	○	○	○	○
	this person went to a traditional healer	○	○	○	○	○	○
	this person self-medicated (i.e., took oral hydration fluid or medicine he or she bought)	○	○	○	○	○	○
	this person isolated him or herself in the bush/forest	○	○	○	○	○	○
(If no Ebola Treatment Center, skip to question 56)	other: ____________	○	○	○	○	○	○
52. Why did this person choose to go to an Ebola Treatment Center? Select all that apply.	because he/she thought it was the right thing to do	○	○	○	○	○	○
	because he/she was afraid he/she was going to die	○	○	○	○	○	○
	because of proximity	○	○	○	○	○	○
	because the cost of care would be covered	○	○	○	○	○	○
	because of the medical resources available there	○	○	○	○	○	○
	because of the expertise of the Ebola Treatment Center staff	○	○	○	○	○	○
	because he/she trusted the Ebola Treatment Center	○	○	○	○	○	○
	because there was no clinical care available near where he/she lived	○	○	○	○	○	○
	because he/she was taken there without being asked	○	○	○	○	○	○
	because the media suggested he/she go to the nearest health facility	○	○	○	○	○	○
	other: ____________	○	○	○	○	○	○

590399-402351

Sick Person Matrix

Participant ID

____

(For reference only)

		person 1	person 2	person 3	person 4	person 5	person 6
53. Where was that Ebola Treatment Center located or what was the name? (Name, city/town/zone)		______ ______	______ ______	______ ______	______ ______	______ ______	______ ______
54. How many days was this person sick before he/she went to the Ebola Treatment Center?	days	☐	☐	☐	☐	☐	☐
	don't know	○	○	○	○	○	○
55. When did this person leave the Ebola Treatment Center?	date	______	______	______	______	______	______
Skip to question 57	don't know	○	○	○	○	○	○
56. If no, why did this person chose NOT to go to an Ebola Treatment Center? Select all that apply.							
	because of the distance	○	○	○	○	○	○
	because he/she thought he/she wasn't very sick	○	○	○	○	○	○
	because he/she didn't think they had the resources needed	○	○	○	○	○	○
	because he/she didn't think they had the necessary expertise	○	○	○	○	○	○
	because he/she did not trust the Ebola Treatment Center	○	○	○	○	○	○
	he/she was told there was no bed available	○	○	○	○	○	○
	because he/she was afraid of getting in the ambulance/fear that it was contaminated	○	○	○	○	○	○
	because he/she was afraid of getting sick with Ebola at the Ebola Treatment Center	○	○	○	○	○	○
	fear of being stigmatized	○	○	○	○	○	○
	he/she was afraid his/her family would be quarantined	○	○	○	○	○	○
	fear of dying at the Ebola Treatment Center	○	○	○	○	○	○
	fear of leaving his/her family	○	○	○	○	○	○
	other: ______________	○	○	○	○	○	○
57. Did this person survive Ebola?	yes	○	○	○	○	○	○
	no	○	○	○	○	○	○
58. While someone was sick at your home with Ebola, what (if anything) did you do to try to avoid getting sick with Ebola? Ask 'Anything else?'		______ (8 lines)	______ (8 lines)	______ (8 lines)	______ (8 lines)	______ (8 lines)	______ (8 lines)

590400-402351

Participant ID

____

(For reference only)

City Section

In this section, I'm going to ask you about things that happened in your city/town/zone during the Ebola outbreak, that is from 1 June 2013 through 31 March 2016.

59. In your opinion, what do you think caused the Ebola outbreak here in your country? Select all that apply.
   - ○ contact with sick people and/or their bodily fluids
   - ○ contact with wild animals
   - ○ contact with domestic animals and/or excretia
   - ○ bad food or water
   - ○ bad spirits/witchcraft
   - ○ wound or injury
   - ○ contact with a corpse/dead body
   - ○ I don't know
   - ○ or something else? Specify: ______________

The next question is about quarantine activities during the Ebola outbreak. It is not always possible to follow quarantine procedures.

(IF NO QUARANTINE ACTIVITY, SKIP TO QUESTION 61)

60. During the Ebola outbreak, that is from 1 June 2013 through 31 March 2016, did you leave quarantine for any reason?
   - ○ yes If yes, why?______________
   - ○ no
   - ○ N/A

The following questions are about the impact of Ebola on your community.

(IF NO EBOLA CASES, SKIP TO ANIMAL CONTACT SECTION)

61. During the Ebola outbreak, that is from 1 June 2013 through 31 March 2016, where did (other community)people (besides you) go for health care?
   - ○ clinic/health center
   - ○ hospital
   - ○ mobile clinic
   - ○ community health worker
   - ○ traditional healer
   - ○ dispensary or pharmacy
   - ○ Ebola Treatment Center
   - ○ other: ______________
   - ○ nowhere
   - ○ do not use

62. During the Ebola outbreak, that is from 1 June 2013 through 31 March 2016, were any animals interacting with dead bodies around Ebola burial sites?
   - ○ yes If yes, which taxa?
   - ○ no
   - ○ don't know

   - ○ rodents/shrews
   - ○ bats
   - ○ non-human primates
   - ○ birds
   - ○ carnivores
   - ○ ungulates
   - ○ pangolins
   - ○ do not use
   - ○ poultry/other fowl
   - ○ goats/sheep
   - ○ camels
   - ○ swine
   - ○ cattle/buffalo
   - ○ dogs
   - ○ cats
   - ○ I don't know

63. During the Ebola outbreak, that is from 1 June 2013 through 31 March 2016, when people in your community were ill with Ebola, did animals ever come into contact with the human excrement, blood, urine, or other bodily fluids of those sick with Ebola?
   - ○ yes If yes, which taxa?
   - ○ no
   - ○ don't know

   - ○ rodents/shrews
   - ○ bats
   - ○ non-human primates
   - ○ birds
   - ○ carnivores
   - ○ ungulates
   - ○ pangolins
   - ○ poultry/other fowl
   - ○ goats/sheep
   - ○ camels
   - ○ swine
   - ○ cattle/buffalo
   - ○ dogs
   - ○ cats

590401-402351

Participant ID ____________________
(For reference only)

64. During the Ebola outbreak, that is from 1 June 2013 through 31 March 2016, were there times that you or people around you did not have enough food to eat?
- ○ yes
- ○ no

(If no, skip to Animal Contact Section)

65. During the Ebola outbreak, that is from 1 June 2013 through 31 March 2016, were animals killed and eaten to help deal with the food shortage?
- ○ yes
- ○ no

If yes, which taxa?

- ○ rodents/shrews
- ○ bats
- ○ non-human primates
- ○ birds
- ○ carnivores
- ○ ungulates
- ○ pangolins
- ○ poultry/other fowl
- ○ goats/sheep
- ○ camels
- ○ swine
- ○ cattle/buffalo
- ○ dogs
- ○ cats

Participant ID

(For reference only)

Animal Contact

In this section, I'm going to ask you about the animals in your life during the Ebola outbreak, between 1 June 2013 through 31 March 2016

66. Did anyone you live with have an animal as a pet?
    - ◯ yes
    - ◯ no

    If yes, which taxa? (Select all applicable taxa)
    - ◯ rodents/shrews
    - ◯ bats
    - ◯ non-human primates
    - ◯ birds
    - ◯ carnivores
    - ◯ ungulates
    - ◯ pangolins
    - ◯ poultry/other fowl
    - ◯ goats/sheep
    - ◯ camels
    - ◯ swine
    - ◯ cattle/buffalo
    - ◯ dogs
    - ◯ cats

67. Did you handle live animals?
    - ◯ yes
    - ◯ no

    If yes, which taxa? (Select all applicable taxa)
    - ◯ rodents/shrews
    - ◯ bats
    - ◯ non-human primates
    - ◯ birds
    - ◯ carnivores
    - ◯ ungulates
    - ◯ pangolins
    - ◯ poultry/other fowl
    - ◯ goats/sheep
    - ◯ camels
    - ◯ swine
    - ◯ cattle/buffalo
    - ◯ dogs
    - ◯ cats

68. Did you raise live animals?
    - ◯ yes
    - ◯ no

    If yes, which taxa? (Select all applicable taxa)
    - ◯ rodents/shrews
    - ◯ bats
    - ◯ non-human primates
    - ◯ birds
    - ◯ carnivores
    - ◯ ungulates
    - ◯ pangolins
    - ◯ poultry/other fowl
    - ◯ goats/sheep
    - ◯ camels
    - ◯ swine
    - ◯ cattle/buffalo
    - ◯ dogs
    - ◯ cats

69. Did you share a water source with animals for washing?
    - ◯ yes
    - ◯ no

    If yes, which taxa? (Select all applicable taxa)
    - ◯ rodents/shrews
    - ◯ bats
    - ◯ non-human primates
    - ◯ birds
    - ◯ carnivores
    - ◯ ungulates
    - ◯ pangolins
    - ◯ poultry/other fowl
    - ◯ goats/sheep
    - ◯ camels
    - ◯ swine
    - ◯ cattle/buffalo
    - ◯ dogs
    - ◯ cats

70. Did you see animal feces in or near food before eating?
    - ◯ yes
    - ◯ no

    If yes, which taxa? (Select all applicable taxa)
    - ◯ rodents/shrews
    - ◯ bats
    - ◯ non-human primates
    - ◯ birds
    - ◯ carnivores
    - ◯ ungulates
    - ◯ pangolins
    - ◯ poultry/other fowl
    - ◯ goats/sheep
    - ◯ camels
    - ◯ swine
    - ◯ cattle/buffalo
    - ◯ dogs
    - ◯ cats

71. Did you eat food after an animal had touched or damaged it? (For example, chew marks or scratches.)
    - ◯ yes
    - ◯ no

    If yes, which taxa? (Select all applicable taxa)
    - ◯ rodents/shrews
    - ◯ bats
    - ◯ non-human primates
    - ◯ birds
    - ◯ carnivores
    - ◯ ungulates
    - ◯ pangolins
    - ◯ poultry/other fowl
    - ◯ goats/sheep
    - ◯ camels
    - ◯ swine
    - ◯ cattle/buffalo
    - ◯ dogs
    - ◯ cats

590403-402351

Participant ID ____________
(For reference only)

72. Did any animals come inside the dwelling where you live?
- ○ yes
- ○ no

If yes, which taxa? (Select all applicable taxa)

○ rodents/shrews	○ poultry/other fowl
○ bats	○ goats/sheep
○ non-human primates	○ camels
○ birds	○ swine
○ carnivores	○ cattle/buffalo
○ ungulates	○ dogs
○ pangolins	○ cats

73. Did you cook or handle meat, organs, or blood from a recently killed animal?
- ○ yes
- ○ no

If yes, which taxa? (Select all applicable taxa)

○ rodents/shrews	○ poultry/other fowl
○ bats	○ goats/sheep
○ non-human primates	○ camels
○ birds	○ swine
○ carnivores	○ cattle/buffalo
○ ungulates	○ dogs
○ pangolins	○ cats

74. Did you eat raw, undercooked, or smoked meat, organs or blood?
- ○ yes
- ○ no

If yes, which taxa? (Select all applicable taxa)

○ rodents/shrews	○ poultry/other fowl
○ bats	○ goats/sheep
○ non-human primates	○ camels
○ birds	○ swine
○ carnivores	○ cattle/buffalo
○ ungulates	○ dogs
○ pangolins	○ cats

75. Did you eat an animal that you knew was sick?
- ○ yes
- ○ no

If yes, which taxa? (Select all applicable taxa)

○ rodents/shrews	○ poultry/other fowl
○ bats	○ goats/sheep
○ non-human primates	○ camels
○ birds	○ swine
○ carnivores	○ cattle/buffalo
○ ungulates	○ dogs
○ pangolins	○ cats

76. Did you find a dead animal and collect it to eat, share, or sell?
- ○ no
- ○ yes, eat
- ○ yes, share
- ○ yes, sell

If yes to any, which taxa? (Select all applicable taxa)

○ rodents/shrews	○ poultry/other fowl
○ bats	○ goats/sheep
○ non-human primates	○ camels
○ birds	○ swine
○ carnivores	○ cattle/buffalo
○ ungulates	○ dogs
○ pangolins	○ cats

77. Were you scratched or bitten by an animal?
- ○ yes
- ○ no

If yes, which taxa? (Select all applicable taxa)

○ rodents/shrews	○ poultry/other fowl
○ bats	○ goats/sheep
○ non-human primates	○ camels
○ birds	○ swine
○ carnivores	○ cattle/buffalo
○ ungulates	○ dogs
○ pangolins	○ cats

590404-402351

Participant ID

(For reference only)

78. Did you slaughter an animal?
- yes
- no

If yes, which taxa? (Select all applicable taxa)
- rodents/shrews
- bats
- non-human primates
- birds
- carnivores
- ungulates
- pangolins
- poultry/other fowl
- goats/sheep
- camels
- swine
- cattle/buffalo
- dogs
- cats

79. Did you hunt or trap an animal?
- yes
- no

If yes, which taxa? (Select all applicable taxa)
- rodents/shrews
- bats
- non-human primates
- birds
- carnivores
- ungulates
- pangolins
- poultry/other fowl
- goats/sheep
- camels
- swine
- cattle/buffalo
- dogs
- cats

80. During the Ebola outbreak, that is from 1 June 2013 through 31 March 2016, the last time you were scratched or bitten, or cut yourself while butchering or slaughtering, what did you do?
Do not read, but select all that apply.
- let someone else take over
- wash wound with soap and water
- rinse wound with water
- bandage wound
- visit doctor
- nothing - kept working
- never butcher or slaughter
- N/A

81. Before the Ebola outbreak, that is before 1 June 2013, were you worried about disease or disease outbreaks in live animals in the local market/area?
- yes
- no

82. During the Ebola outbreak, that is from 1 June 2013 through 31 March 2016, were you worried about diseases or disease outbreaks in live animals in the local market/area?
- yes
- no

83. Since the Ebola outbreak, that is after 31 March 2016, have you been worried about diseases or disease outbreaks in live animals in the local market/area?
- yes
- no

84. During the Ebola outbreak, that is from 1 June 2013 through 31 March 2016, what were the activities you did to earn your livelihood?
Select all that apply.
- extraction of minerals, gas, oil timber
- crop production
- wildlife restaurant business
- wild/exotic animal trade/market business
- rancher/farmer animal production business
- meat processing, slaughterhouse, abattoir
- zoo/sanctuary animal health care
- protected area worker
- hunter/trapper/fisher
- forager/gatherer/non-timber forest product collector
- migrant laborer
- nurse, doctor, traditional healer, community health worker
- construction (road, housing)
- other: ____________________

Participant ID
____
(For reference only)

85. During the Ebola outbreak, that is from 1 June 2013 through 31 March 2016, was there a significant change to your activities?

Select all that apply.
- ◯ no change (Skip to End of Section for additional module directions.)
- ◯ activities stopped
- ◯ activities decreased
- ◯ activities increased

86. For how long were your activities interrupted? ______________ in weeks

Additional Module Instructions

If domestic animal production or meat processing on Q84, conduct ANIMAL PRODUCTION Module

If hunter/trapper/fisher on Q84 OR Yes to Q79, conduct HUNTER Module

590406-402351

Ebola Host Project PREDICT
Human Questionnaire

EIDITH
V 1.1

Add Site and Event Form ID: 0 1 2 3 4 5 6 7 8 9 / 0 1 2 3 4 5 6 7 8 9 / 0 1 2 3 4 5 6 7 8 9 / 0 1 2 3 4 5 6 7 8 9 / 0 1 2 3 4 5 6 7 8 9 / 0 1 2 3 4 5 6 7 8 9

Site name: ______________________

(For reference only)

1. Participant ID: ______________________ Consent Form Administered & Signed ◯ yes ◯ no

2. Description of Interview Location - Select all that apply.
(To be completed by interviewer prior to administrative questionnaire. Prepare and download modules in advance.)
   - ◯ Animal Production or Abattoir Site
   - ◯ Crop Production Site
   - ◯ Extractive Industry Site
   - ◯ Market or Value Chain Site
   - ◯ Temporary Settlement Site
   - ◯ Natural Areas (eg. forest, urban park/garden)
   - ◯ Wildlife Restaurant
   - ◯ Zoos or Sanctuaries
   - ◯ Outbreak Investigation Site
   - ◯ Control Site
   - ◯ Other: ______________________

3. Date of interview ______________________

4. Begin time of interview ________________
(Example: 17:50)

5. End time of interview ________________
(Example: 19:20)

6. Where are you conducting this interview?

Village/Town/City ______________ Province/State ______________

Latitude ______________________ Longitude ______________________

Interviewer: Please collect GPS coordinates if administering using paper and pen.

7. Interviewer Observed Gender ◯ male ◯ female ◯ other

INTERVIEW/QUESTIONNAIRE BEGINS

Demographics Section (include observation question 7)

8. How old are you? ____________
If the exact age is unknown, enter the respondent's estimated age.

9. Where do you live?

Village/Town/City ______________ Province/State ______________

Latitude ______________________ Longitude ______________________

Interviewer: Probe for landmarks or nearest known site if area unknown.
GPS coordinates to be identified and entered after completion of interview.

586893-456668

10. How long have you lived there?
    Select one option.
    - ◯ <1 month
    - ◯ 1 month - 1 year
    - ◯ >1 - 5 years
    - ◯ >5 - 10 years
    - ◯ >10 years

11. How many other people live in the dwelling where you live? ________
    Skip to question 14 if answer is 0.

12. How many in the dwelling are children less than 5 years old? ________

13. How many in the dwelling are male? ________

14. How many rooms are there in the dwelling where you live? ________
    (Do not include bathroom or kitchen)

15. Is the dwelling a permanent structure (that cannot be moved)?
    - ◯ yes
    - ◯ no

Participant ID ____________ (For reference only)

Livelihood Section

In this section, I'd like to ask you about education and the kinds of work activities that you have done since this time last year.

16. What is the highest level of education you have completed? Select one option.
- primary school
- secondary school
- college/university/professional
- none

17. What is the highest level of education that your mother completed? Select one option.
- primary school
- secondary school
- college/university/professional
- none

18. Since this time last year what are the activities you have done to earn your livelihood? Select all that apply
- extraction of minerals, gas, oil timber
- crop production
- wildlife restaurant business
- wild/exotic animal trade/market business
- rancher/farmer animal production business
- meat processing, slaughterhouse, abattoir
- zoo/sanctuary animal health care
- protected area worker
- hunter/trapper/fisher
- forager/gatherer/non-timber forest product collector
- migrant laborer
- nurse, doctor, traditional healer, community health worker
- construction (road, housing)
- other: ____________

19. If more than one activity was selected, what is the activity on which you spend the most time since this time last year? Select one option.
- extraction of minerals, gas, oil timber
- crop production
- wildlife restaurant business
- wild/exotic animal trade/market business
- rancher/farmer animal production business
- meat processing, slaughterhouse, abattoir
- zoo/sanctuary animal health care
- protected area worker
- hunter/trapper/fisher
- forager/gatherer/non-timber forest product collector
- migrant laborer
- nurse, doctor, traditional healer, community health worker
- construction (road, housing)
- other: ____________

20. Which best describes your job position? Select one option.
- manager/owner/foreman
- worker
- live and work at home independently (If chosen, skip to Medical History Section.)
- professional
- other: ____________

21. Where do you work?

Village/Town/City ____________ Province/State ____________

Latitude ____________ Longitude ____________

Interviewer: Probe for landmarks or nearest known site if area unknown.
GPS coordinates to be identified and entered after completion of interview.

586895-456668

Participant ID ______________
(For reference only)

Medical History Section

In this section, I'm going to ask you about illness and treatment that have occurred in the community.

22. Before the Ebola outbreak, that is before 1 June 2013, where did you usually get treatment for medical problems? Select all that apply.
   - ○ clinic/health center
   - ○ hospital
   - ○ mobile clinic
   - ○ community health worker
   - ○ traditional healer
   - ○ dispensary or pharmacy

23. During the Ebola outbreak, that is from 1 June 2013 through 31 March 2016, where did you usually get treatment for medical problems? Select all that apply.
   - ○ clinic/health center
   - ○ hospital
   - ○ mobile clinic
   - ○ community health worker
   - ○ traditional healer
   - ○ dispensary or pharmacy

24. During the Ebola outbreak, that is from 1 June 2013 through 31 March 2016, did you have an unusual illness with any of the following symptoms? Select all that apply. (READ ONLY SYMPTOMS)
   - ○ fever with headache and severe fatigue or weakness (encephalitis)
   - ○ fever with bleeding or bruising not related to injury (hemorrhagic fever)
   - ○ fever with cough and shortness of breath or difficulty breathing (SARI)
   - ○ fever with muscle aches, cough, or sore throat (ILI)
   - ○ fever with diarrhea or vomiting
   - ○ fever with rash
   - ○ persistent rash or sores on skin
   - ○ no (if no, skip to question 26)
   - ○ yes, but none of these symptoms-describe: ______________

25. In your opinion, when you were sick, what caused this sickness? Select all that apply.
   - ○ contact with sick people and/or their bodily fluids
   - ○ contact with wild animals
   - ○ contact with domestic animals and/or excretia
   - ○ bad food or water
   - ○ bad spirits/witchcraft
   - ○ wound or injury
   - ○ contact with a corpse/dead body
   - ○ I don't know
   - ○ other: ______________

26. During the Ebola outbreak, that is from 1 June 2013 through 31 March 2016, were you diagnosed with Ebola?
   - ○ yes
   - ○ no

If No to question 24 & No to question 26, Skip to question 42
If No to question 26 & Yes to question 24, Skip to question 28

27. In your opinion, when you were sick with Ebola, what caused this sickness? Select all that apply.
   - ○ contact with sick people and/or their bodily fluids
   - ○ contact with wild animals
   - ○ contact with domestic animals and/or excretia
   - ○ bad food or water
   - ○ bad spirits/witchcraft
   - ○ wound or injury
   - ○ contact with a corpse/dead body
   - ○ I don't know
   - ○ other: ______________

28. When did you first become sick with Ebola (or that unusual illness)? ______________ date
   I don't remember the date. ○

586896-456668

Ebola Host Project PREDICT Hunter Module

EIDITH

V 1.0

0	1	2	3	4	5	6	7	8	9
0	1	2	3	4	5	6	7	8	9
0	1	2	3	4	5	6	7	8	9
0	1	2	3	4	5	6	7	8	9
0	1	2	3	4	5	6	7	8	9
0	1	2	3	4	5	6	7	8	9

Add Human Questionnaire Form ID

Participant ID ____________________

(For reference only)

1. During the Ebola outbreak, that is from 1 June 2013 through 31 March 2016, what animals did you hunt?
   Select all that apply.
   - ○ rodents/shrews
   - ○ bats
   - ○ non-human primates
   - ○ birds
   - ○ carnivores
   - ○ ungulates
   - ○ pangolins

2. During the Ebola outbreak, that is from 1 June 2013 through 31 March 2016, what methods did you use to hunt/trap animals?
   Select all that apply.
   - ○ snare
   - ○ bow
   - ○ hands
   - ○ gun
   - ○ machete
   - ○ knife
   - ○ net
   - ○ cage
   - ○ trap
   - ○ other:________________

3. During the Ebola outbreak, that is from 1 June 2013 through 31 March 2016, what was the purpose of your trapping or hunting?
   Select all that apply.

	for consumption at home	for use of animal products at home	for sale for consump -tion	for sale alive at market	for sale of animal products	live trapping of nuisance animals for translocation	culling of nuisance animals
rodents/shrews	○	○	○	○	○	○	○
bats	○	○	○	○	○	○	○
non-human primates	○	○	○	○	○	○	○
birds	○	○	○	○	○	○	○
carnivores	○	○	○	○	○	○	○
ungulates	○	○	○	○	○	○	○
pangolins	○	○	○	○	○	○	○

During the Ebola outbreak, that is from 1 June 2013 through 31 March 2016, when you hunted or trapped:

4. Were you exposed to blood?
   - ○ yes
   - ○ no

5. Were you scratched or bitten?
   - ○ yes
   - ○ no

6. During the Ebola outbreak, that is from 1 June 2013 through 31 March 2016, did you see an outbreak of dead wild animals?
   - ○ yes
   - ○ no

7. If yes, which wild animals?
   Select all that apply.
   - ○ rodents/shrews
   - ○ bats
   - ○ non-human primates
   - ○ birds
   - ○ carnivores
   - ○ ungulates
   - ○ pangolins

472981-643072

8. During the Ebola outbreak, that is from 1 June 2013 through 31 March 2016, what did you do when you found an animal dead (not in a trap or shot by another hunter)?
Select all that apply.
   - ◯ touch it to see if it is still fresh
   - ◯ butcher in the forest
   - ◯ smoke or cook in the forest
   - ◯ take home to prepare
   - ◯ bury it
   - ◯ report it to authorities
   - ◯ take it to sell it
   - ◯ nothing
   - ◯ other: ________________

9. During the Ebola outbreak, that is from 1 June 2013 through 31 March 2016, how did you transport a dead animal, if you took it?
Select all that apply.
   - ◯ not wrapped
   - ◯ wrapped in leaves or other natural material
   - ◯ wrapped in plastic
   - ◯ in a bag
   - ◯ in a basket

10. During the Ebola outbreak, that is from 1 June 2013 through 31 March 2016, did you have special protective equipment (eg. shoes, masks, gloves)?
   - ◯ yes
   - ◯ no

11. If yes, which protective equipment?
Select all that apply.
   - ◯ shoes/boots
   - ◯ mask
   - ◯ clothes
   - ◯ gloves
   - ◯ gown/apron

12. During the Ebola outbreak, that is from 1 June 2013 through 31 March 2016, when did you use protective equipment?
Select all that apply.
   - ◯ handling animals
   - ◯ slaughter
   - ◯ butcher
   - ◯ always on at work
   - ◯ other: ________________
