# Supplementary material for: Socializing One Health: an innovative strategy to investigate social and behavioral risks of emerging viral threats
Source: One Health Outlook. 2021 May 14;3:11. doi: 10.1186/s42522-021-00036-9 (PMC8122533; doi:10.1186/s42522-021-00036-9)
Supplement: Supplementary file 3 — Additional file 3. Ethnographic interview and focus group discussion guides administered in 13 countries during in-depth qualitative behavioral risk investigations. [file 42522_2021_36_MOESM3_ESM.pdf]

ETHNOGRAPHIC INTERVIEW GUIDE

Core Themes

1. Human movement
2. Socioeconomics
3. Biosecurity in human environments
4. Illness, medical care/treatment and death of humans
5. Human-animal contact

HUMAN MOVEMENT

GOAL: To understand living environment and 'home range' (e.g., how far people travel and why).

Home

Where do you live/what kind of dwelling? How many people are in the household? How many rooms? How many are children? Is everyone related? Sleeping arrangements?

How often do you move? Any seasonality of movements?—eg, for work, for food, for safety (e.g., against flood, drought, conflict)?

What are the things you do to protect your home (against predators, animals, outsiders, bad weather)?

Work

What kind of work or activities do you do? What do other household members do? Where do these activities happen?

How do you protect your activities and business interests? (e.g., grazing or crop land, business competition, hunting territory, animal stock)

Travel

How far do household members travel from home and why? (Follow up on animal related issues: shopping, selling/buying/trading, hunting, transport, etc)

How travel (by foot, bike, cart, truck, plane)? Is it ever for overnight? Where stay?

Why traveling? (work/migrant, family, religion, holidays, to sell/trade/buy animals)

Other family members in other areas of the country? Visit often?

Observed environment

Have there been any changes in the environment: new roads, more boats or ports, fields, buildings, population movement (in or out), land clearing or abandonment, new houses, other new buildings

Who is responsible for the changes? Are the changes good or bad?

SOCIOECONOMICS

GOAL: To understand a typical day and how money and social standing impact opportunity and risk.

Daily routine

Tell me about your daily routine (get description of work on a usual day, include purchasing and preparing food, timing of types of meals, responsibilities/duties related to animals, any changes by season)

How do people in the household contribute to earning money and getting food (and water)?

Where do the children play? Who takes care of the children when you are at work?

Animal responsibilities

Describe the animal related jobs and responsibilities for people at every age (i.e., young children, older children, young adults, adults, elderly).

What are the skills/knowledge needed before moving to the next stage of duties/responsibilities?

Are there differences in responsibilities between boys and girls, men and women, by ethnicity or class?

Education

How many children are currently in school? Until what age do your children go to school? (boys and girls?)

What is your level of education? Why did you stop?

Economics

Do you make more money than other people who do the same things as you? Why do you think that is?

Are there times of year when you make less money? What happens then?
Are there times when food is more expensive than others? Tell me about that (eg, different food availability, seasonal, festival related).
Do you think you and your household are better off than most people? Could you do things to make it better?

BIOSECURITY IN HUMAN ENVIRONMENTS
GOAL: To determine if any sanitation or hygiene factors could play a role in disease spillover
Water and food
Is there a central source of water? What is the source? (eg, pond, uncovered well, rainwater, taps, covered well)
Is there a water source you like better?
How far away is the water source? Do animals drink from the same source?
Do you do anything to your drinking water to clean it before you drink it?
How do you store your food? (e.g., open containers, covered, hanging, refrigerate)
Do you eat or drink things where you suspect animal contact? (e.g., teeth/scratch marks, feces or urine seen)
Do you regularly clean your food prep station/kitchen and tools? How?
Sanitation
Are there toilets, latrines or other designated areas for human waste? Are these cleaned and used regularly?
Are butchering and slaughtering areas separate? How often are they cleaned and how? Who does the cleaning?
Are there any official rules or laws about human waste and garbage disposal?
Are there any animal pest control laws? What do you do to control animal pests?
Hygiene
When are the best times to wash your hands? Do you use soap? How much does soap cost and where get it?
Do you wash your hands at home? at work?
How often and where do you and your household members bathe?

ILLNESS, MEDICAL CARE/TREATMENT, DEATH
GOAL: To identify any unusual disease experiences—signs, symptoms and sources
Household illness
Is anyone sick right now?
What do you do when someone in the household gets sick? Who takes care of that person?
The last time someone was seriously sick what happened (explore when, with what, how did they get sick, who told/consulted, anyone else get sick after, final outcome)?
Has anyone ever had an sickness that people don't usually get? What happened? Where did it come from?
Illness from animals
Do you know anyone who has gotten sick from an animal? What animal? What did they get? What happened?
Do you know any other diseases/illnesses people can get from animals? How does the animal give the illness to the person? How often does it happen?
Medical care/treatment
How sick would you have to feel to stay home and not do normal routine?
Where do you go when you are sick?
Do you prefer to use traditional medicine, western medicine or a combination?
How sick would you have to feel to go to doctor/clinic/hospital? What does that cost? (in time, lost wages/business, transport costs, etc) How far away?
Death
What is the tradition when someone dies? (Explore if reported to authorities, differ by age or gender, what happens to the body, does the community come together or is it private.)

HUMAN ANIMAL CONTACT

GOAL: To gain knowledge about interactions with animals, animal health and animal perceptions and knowledge.

Encourage but don't lead discussion about which animals. Allow respondent to name the animals. If no birds or bats are mentioned, follow up by asking specific questions about birds and bats.

<u>Indirect contact</u>

What kind of meat do people in your household eat? How do you get it/where does it come from? What is furthest away an animal comes from?

Is meat dead or alive when you get it? If dead(/prepared), how to tell if good/fresh?

If alive, how long are live animals kept before being sold or eaten? How do you get live animals home?

How is meat prepared (raw/undercooked)? Is meat prepared in the same place as other activities? (e.g., preparing vegetables, cleaning babies/changing diapers, where other food or drinking water is stored)

Do animals come in or near the dwelling? How do you know animals are there? Which animals?

<u>Direct contact</u>

Do you or someone in your household handle live animals? In what context? (e.g. ranching/animal husbandry, hunting, wet markets, work, around dwelling/other building, pets)

What are the animals that you keep/raise or sell? How many different kinds of animals? How many of each? For how long do you have the animals?

Where do live animals come from? Where is the furthest away an animal comes from?

Who buys/trades for your live animals? Where do the animals go?

Have you been bitten, scratched or had bleeding after handling an animal? By a wild animal?

Where are live animals slaughtered? butchered? Do people buy or sell parts?

Do you travel with animals? Explore details of the process, specific routes and encounters (eg, with other animals, with animal transport supporting industries, such as holding areas, restaurants, hotels) along the way.

Explore for differences over time in animal handling, eg, seasonality, legal, religious, animal reproduction

<u>Animal products/rituals</u>

Other uses of animals—e.g., as pets, medicine, magic, fertilizer*,* for trading

Rules for children around wild animals as pets, playing with wild animals or dead animals

<u>Animal health</u>

How do you care for your animals: how are they fed, what do they eat, where do they eat/graze and sleep? Are they segregated or all together? Differences by season? day/night? Does anyone live or stay with the animals? Is there a central area for animal waste? How often are animal cages, stalls, or penned areas cleaned? Who cleans them?

Do the animals get veterinary care? Vaccinations?

How do you know when an animal is sick? What's the first thing you do about a sick animal?

Have you seen an animal outbreak or die-off? What happened?

<u>Perceptions and knowledge</u>

What are the most unusual animals anyone can buy?—seasonal? Expensive? Who buys?

Are there any animals you avoid eating? Why? Ever heard of anyone eating/selling dead or infected animals?

Do people ever eat non-domesticated animals/wildlife? Where do they get them?

Who usually buys wildlife products? Have there been changes over time?

What do you do when you find a dead animal?

What laws about animals do you know? (eg, limiting/outlawing hunting, reporting and culling of sick animals)

FOCUS GROUP GUIDE Version 2, May 1, 2015

The focus group discussion is initiated by naming all of the animals that can be found in the community. The goal of this exercise is to explore animal diversity.

The community mapping activity locates where the different kinds of animals can be found relative to the site of the focus group. It should be emphasized that this will not be an 'accurate' map. This exercise is designed to assess the distribution and overlap of animals. Prompts such as 'anywhere else?' should be used. The animal list will contain insects, reptiles and fish. ***Map only mammalian and avian species***.

These two activities together should be limited to 10-15 minutes. The themes to be explored in the discussion are 1) contact and context, 2) illness in animals and humans, and 3) rules and restrictions. Events such as animal die-offs should be added to the map, if they are discussed.

1) Contact and context

- Which of these animals do you see the most often? The least? (Probe: where, why)
- What animals do you come into physical contact with? (Probe: where, why, how often)
- Which of these animals do you eat?
    - Where do you get them? How are they prepared? Which are for special occasions only?
- What are animals good for other than food? (probe: labor, medicinal, magic, pets, by-product uses)
- Which animals come into buildings or places where people are? Is water shared with animals?
- How are unwanted animals kept out? (probe: which animals, all methods used)
- Who takes care of the animals? (Probe: who, specific jobs, animal movements)

2) Illness in animals and humans

Animals

- What happens when animals get really sick? How are the animals cared for?
- Has this happened recently? Do people try to hide animal sickness?
- Is animal sickness reported to anyone? (probe for differences between wild and domestic animals)
- Have any animals been destroyed or killed by authorities? Describe.
- What happens to animals when they die? (probe: eaten, buried, left to rot, depends if wild or not)

Humans

- What is the most unusual or memorable sickness anyone has had? What happened?
- What are the causes of illness or sickness?
- Do you know anyone who has gotten sick from an animal? What happened?
- What do you know about animals that can give you infections or diseases?

3) Rules and restrictions

- Are there places in the community where you aren't allowed to go? Why not?
- Are there any rules about hunting or trapping animals? (Probe: cultural, legal)
- Are there any animals that you don't eat or that are avoided? Why?
- Are there official rules or laws about garbage disposal? Human waste? Animal waste?
- Is garbage a problem in this community? What's the problem?

Final question for all: If you could change one thing in your life, what would it be and how would you do it?
